# No extra-adrenal aldosterone production in various human cell lines

**DOI:** 10.1530/JME-23-0100

**Published:** 2024-02-01

**Authors:** Isabelle Durrer, Daniel Ackermann, Rahel Klossner, Michael Grössl, Clarissa Vögel, Therina Du Toit, Bruno Vogt, Heidi Jamin, Markus G Mohaupt, Carine Gennari-Moser

**Affiliations:** 1 Department of Nephrology and Hypertension University of Bern, Berne, Switzerland; 2 Department of Internal Medicine, Sonnenhof, Lindenhofgruppe, Berne, Switzerland; 3 Department for BioMedical Research University of Bern, Berne, Switzerland

**Keywords:** CYP11B2, aldosterone, primary hyperaldosteronism, progesterone, cell lines

## Abstract

Extra-adrenal *de novo* aldosterone (Aldo) production has been described inconsistently. Systematic data based upon state-of-the-art technology including validated controls are sparse. We hypothesized that aldosterone synthase (*CYP11B2*) expression and *de novo* Aldo production are absent in nonadrenal human cell lines, either immortalized cell lines or commercially available primary cell lines, including peripheral blood mononuclear cells (PBMCs) of individuals without and with primary hyperaldosteronism (PA). CYP11B2-transfected COS-7 and endogenous CYP11B2 expressing adrenal H295R cells served as positive controls. Various well-characterized, purchased, immortalized (BeWo, HEK293, HTR-8/SVneo, JEG-3) and primary (HAEC, HLEC, HRGEC, HRMC, HUAEC, HUVEC, PBMC) cell lines as well as self-isolated PBMCs from PA patients (*n* = 5) were incubated with the steroid hormone substrates progesterone, deoxycorticosterone, corticosterone or 18-OH-corticosterone with and without Ang II for 24 h to assess CYP11B2 enzymatic activity. *CYP11B2* expression was analyzed by real-time PCR and liquid chromatography–mass spectrometry was used to quantify Aldo production. Pronounced *CYP11B2* mRNA expression and Aldo production were observed in both positive controls, which followed an incremental time course. Neither substrates alone nor coincubation with Ang II significantly stimulated *CYP11B2* expression or Aldo production in various immortalized and primary cell lines and PBMCs of PA patients. These results strongly support the absence of relevant *de novo* extra-adrenal Aldo production in nonadrenal cells, including blood mononuclear cells, irrespective of the absence or presence of autonomous adrenal Aldo production.

## Introduction

Next to the well-known renal responses, aldosterone (Aldo) has a major impact in nonclassical off-target tissues. This leads to the idea of a local extra-adrenal Aldo synthesis. While during pregnancy, Aldo is beneficial for placental growth (Gennari-Moser *et al.* 2011), it promotes inflammation and fibrosis in vessels and the kidneys. Controversy over local *de novo* Aldo production in these organs explanatory for its adverse effects has started decades ago, while state-of-the-art technology including validated controls might now enable comprehensive investigations.

Extra-adrenal Aldo production has been postulated based upon data by Casey *et al.* in 1982 pointing towards the conversion of plasma progesterone to 11-deoxycorticosterone (DOC) in extra-adrenal tissues (including the kidney, aorta, spleen, and several fetal tissues) in pregnant and nonpregnant women and in men (Casey & MacDonald 1982), though metabolites further downstream of DOC had not been assessed.

Renal cytochrome P450 aldosterone synthase (*CYP11B2*) expression and Aldo production was described in whole kidney tissue, tubular epithelial cells, and mesangial cells (Wu *et al.* 1999, Nishikawa *et al.* 2005, Xue & Siragy 2005).

In the vasculature, *CYP11B2* expression and Aldo production have been found in mesenteric arteries of healthy (Takeda *et al.* 1994, 1995*a*,*b*, Rudolph *et al.* 2000) and spontaneously hypertensive rats (Takeda *et al.* 1997), in human umbilical vein endothelial cells (HUVEC) (Takeda *et al.* 1996), in endothelial and vascular smooth muscle cells of human pulmonary arteries and the aorta of healthy and diseased subjects (Hatakeyama *et al.* 1994, Maron *et al.* 2012, Matsuzawa *et al.* 2013, Alesutan *et al.* 2017). The level of Aldo production and *CYP11B2* expression observed in endothelial and smooth muscle cells approximated 1/50 of that of adrenal cells (Hatakeyama *et al.* 1994). Aldo production in HUVECs was responsive to angiotensin II (Ang II), adrenocorticotropic hormone (ACTH) and potassium (Takeda *et al.* 1996), and was upregulated in human pulmonary artery endothelial cells in hypoxic conditions (Maron *et al.* 2014). Interestingly, the classical pathway of *de novo* Aldo production from cholesterol as substrate was ruled out as no steroidogenic enzymes upstream of CYP11B2 could be detected in endothelial and vascular smooth muscle cells (Hanukoglu 1992). Consequently, Hatakeyama *et al.* suspected that the enzyme system responsible for Aldo production in human vascular cells is different from that found in the adrenal cortex and that vascular Aldo may be synthesized from metabolic intermediates which originate from the circulation (Hatakeyama *et al.* 1996). In clear contrast are findings of absent *CYP11B2* mRNA expression and Aldo biosynthesis in human umbilical veins, and in human pulmonary artery endothelial cells (Ahmad *et al.* 2004) by a group that used a validated protocol developed to detect very low expression levels of *CYP11B2* in subregions of the human brain (Gomez-Sanchez *et al.* 1997).

In 1999, Takeda *et al.* described *CYP11B2* expression in peripheral blood mononuclear cells (PBMCs) of patients with idiopathic hyperaldosteronism (Takeda *et al.* 1999). *CYP11B2* expression was reported to be upregulated in PBMCs of primary hyperaldosteronism (PA) patients as compared to healthy subjects and patients with Aldo-producing adenoma (Miyamori *et al.* 2000). Later, Miura *et al.* added that PBMCs of healthy subjects produce Aldo upon Ang II stimulation (Miura *et al.* 2006).

The existence of *de novo* Aldo production beyond the adrenal glands is still uncertain in most tissues, complicated by issues with respect to control conditions, and despite given methodological improvements over time. A major demand to any study, targeting the proof of absence of a functionally relevant system, is to apply highly sensitive methods.

We therefore hypothesized, that *CYP11B2* expression and *de novo* Aldo production are absent in nonadrenal human cell lines, either immortalized cell lines or commercially available primary cell lines, including PBMCs of individuals with and without PA. Immortalized cell lines used were: BeWo (human choriocarcinoma cells), HEK293 (human embryonic kidney cells), HTR-8/SVneo (human first-trimester trophoblasts) and JEG-3 (human choriocarcinoma cells). Purchased primary cell lines used were: HAEC (human aortic endothelial cells), HLEC (human lymphatic endothelial cells), HRGEC (human renal glomerular endothelial cells), HRMC (human renal mesangial cells), HUAEC (human umbilical artery endothelial cells), HUVEC (human umbilical vein endothelial cells), and PBMCs (peripheral blood mononuclear cells).

Specifically, we aimed to assess *CYP11B2* mRNA expression and to measure Aldo production first in nonstimulatory conditions and second upon Ang II stimulation.

## Materials and methods

### Material and cell lines

Cell culture materials were from Techno Plastic Products AG (Trasadingen, Switzerland). Collagen I coated petri dishes were from Corning, while poly-l-lysine and fibronectin for cell ware coating were from ScienCell (Chemie Brunschwig, Basel, Switzerland).

BeWo (CCL-98), HTR-8/SVneo (CRL-3271), HAEC (PCS-100-011), COS-7 (CRL-1651) and NCI-H295R cells (CRL-2128) were purchased from ATCC. The primary cells HUVEC (#8000), HUAEC (#8010) and HLEC (#2500) were from ScienCell (Chemie Brunschwig, Basel, Switzerland).

The primary cells HRMC (#4200) and HRGEC (#4000) and their corresponding media, MCM (#4201) and ECM (#1001), with the supplements (MsCGS #4252 and ECGS #1052), penicillin/streptomycin (P/S, #0503) and fetal bovine serum (FBS, #0010 and # 0025) were obtained from ScienCell (Chemie Brunschwig, Basel, Switzerland). HRMC were cultured on poly-l-lysine, and HRGEC on fibronectin or collagen I coated plates. HUAEC cells (#8010) were cultured in ECM (#1001) containing the endothelial cell growth supplements (ECGS #1052) also from ScienCell.

HEK293 (human embryonic kidney cells) #CRL-1573 and JEG-3 (a human choriocarcinoma cell line) #HTB-36 cells were from ATCC, and their corresponding media DMEM (# 41965) and McCoy’s (#36600) respectively, were from Gibco. PBMCs (4W-270) of six healthy individuals (four men, two women) were purchased from Lonza, Basel, Switzerland. Method of authentication of cells was short tandem repeat analysis for ATCC, immunofluorescence for ScienCell, and QC testing for Lonza. HUVEC, HTR-8/SV neo, and BeWo cells and PBMCs were cultured in RPMI1640 #21875 (with phenol red) and #11835 (without phenol red) from ThermoFisherScientific (Reinach, Switzerland). H295R cells were cultured in DMEM-F12 #11320033 (with phenol red) and #21041 (without phenol red) from ThermoFisherScientific.

HAEC and HLEC cells grew in the Vascular Cell Basal Medium (PCS-100-030) containing the supplements (PCS-100-041) from ATCC. HLEC were cultured on collagen I-coated plates.

COS-7 cells were cultured in DMEM # 41965 (with phenol red) and #31053 (without phenol red) from Gibco/ThermoFisher Scientific.

FBS, P/S, HEPES, ITS+ Premix and sodium pyruvate were from Gibco/ThermoFisher Scientific if not otherwise stated.

Fugene E2311(Promega), CYP11B2 plasmid #RC215476 was from Origene, and the pCMV_EV plasmid was a gift. OptiMEM #31985 was from ThermoFisher Scientific.

All steroid standards for LC-MS analysis were purchased from Cerillant (UK) or Steraloids, Inc. (Newport, RI, USA).

### Primary hyperaldosteronism patients and healthy controls

PA patients were recruited at our outpatient clinic of the Department of Nephrology and Hypertension, University Hospital of Bern, Switzerland, for the evaluation and treatment of their arterial hypertension. All patients had signs of secondary hypertension due to primary hyperaldosteronism. The diagnosis was made by measuring plasma Aldo and renin levels in lying and standing position. All medication interfering with the renin–angiotensin system was stopped 2 to 3 weeks prior to the measurements. Patients had an elevated Aldo to renin ratio (ARR > 40), an elevated plasma Aldo (PAC ≥10 ng/dL or ≥ 277 pmol/L) or a suppressed renin. A confirmation test was conducted in cases where the Aldo level was lower than 20 ng/dL or 555 pmol/L and in absence of spontaneous hypokalemia.

Exclusion criteria were: no signed informed consent, hypertension from another cause with an AAR <40 and a renin level greater than 2.6 ng/L. Medications interfering with the mineralocorticoid receptor (MR) such as spironolactone, eplerenone or finerenone, pregnancy or liver cirrhosis were also exclusion criteria. Detailed characteristics of the patients with PA and of the healthy subjects providing PBMCs are summarized in Table 1. Additional details of the purchased PBMCs of healthy volunteers are shown in Supplementary Fig. 1 (see the section on supplementary materials given at the end of this article).

**Table 1:** Characteristics of healthy subjects providing PBMCs and of patients with primary aldosteronism.

Parameter	Subject ID					
Healthy subject						
Healthy subject ID	1	2	3	4	5	6
Age (years)	47	21	43	23	21	44
Gender	M	M	F	M	F	M
**Patients with primary aldosteronism**						
Patient ID	1	2	3	4	5	
Age (years)	68	63	51	65	62	
Gender	M	M	F	M	M	
BP (mm Hg)	150/80	165/100	141/89	145/82	169/105	
BMI (kg/m^2^)	30.2	31.6	21.5	27	25	
Creatinine (µmol/L)	55	104	64	80	93	
eGFR (mL/min/1.73 m^2^)	102	100	96	89	76	
Renin (ng/L)	<1.2	<1.2	<1.2	<1.2	<1.2	
ARR (pmol/ng)	205	133	1000	250	967	
Aldo (pmol/L)	354	236	1240	299	1160	

Aldo, aldosterone; ARR, aldosterone-to-renin ratio; BMI, body mass index; BP, blood pressure; eGFR, estimated glomerular filtration rate.

Clinical work-up of PA patients was done according to standard protocols, full blood was collected, PBMCs were isolated in house and analysis was performed prospectively.

All parts of the studies were approved by the ethics committee of the Canton of Berne, as required for the sample collection according to the Declaration of Helsinki. All patients and participants were only included in the study after signing informed consent.

The provider of the PBMCs from healthy volunteers does not state BMI and BP data.

### Treatment of cells

Cells were cultured in their corresponding media with the lowest amount of FBS necessary to guarantee optimal surviving conditions.

Primary, not terminally differentiated cells (HUVEC, HUAEC, HAEC, HLEC, HRGEC, HRMC) were allowed to double maximum ten times before experiments were performed.

### Transfection of COS-7 cells with CYP11B2 plasmid

CYP11B2 (0.5 µg/well) and an empty plasmid (0.5 µg/well) were mixed with OptiMEM and Fugene. After 15min at RT, the mix was added to cells. Following a 32 h incubation period the cells were washed and serum-free, DMEM was added with the steroid hormone substrates progesterone, DOC, corticosterone or 18-OH-corticosterone at a concentration of 10^−6^ M and with or without AngII (10^−6^ M). After 24 h, the supernatant was collected for LC-MS analysis and total RNA extraction was performed using the TRIzol method.

### Real-time PCR

Cells were cultured for 24 h in a steroid-free and phenol red-free medium alternative with or without Ang II (10^−6^ M). PBS was the solvent of Ang II and served as the baseline.

Extraction of total RNA was performed using the Trizol method. RNA was reverse transcribed by using oligo dT and random hexamer in the same reaction (PrimeScript RT reagent Kit from Takara). All RT experiments in all cell lines were performed the same way. Fifty nanograms of cDNA were used for real-time PCR. Assay on demand primers were used for human CYP11B2 (Hs01597732_m1), SRD5A1 (Hs 00971645_g1), CYP21A2 (Hs 00416901_g1), AGTR1 (Hs00258938_m1), AGTR2 (Hs02621316_s1), cyclophilin A (*PPIA*, 4326316E), and 18S (4310893E). Cyclophilin A and 18S served as endogenous controls. They all were from Applied Biosystems (ThermoFisher Scientific). GoTaq Probe qPCR Master Mix A6102 was from Promega AG.

H295R and COS-7 cells transfected with CYP11B2 were used as positive controls. Results are displayed as ct values. Amplification cycle number was 50 and assays were performed in triplicate.

7500 Fast Real-time PCR and Quant Studio 1 machine were used both for all cell lines assessed. They were from Applied Biosystems (ThermoFisher Scientific).

### Liquid chromatography–mass spectrometry

Cells were cultured for 24 h in a steroid-free and phenol red-free medium alternative with the steroid hormone substrates progesterone, DOC, corticosterone or 18-OH-corticosterone at a concentration of 10^−6^ M and with or without Ang II (10^−6^). EtOH was the solvent of the substrates and served as the baseline. Reasons for phenol red-free medium were to exclude stimulatory conditions and interference of phenol red with the LC-MS equipment. After 24h cell supernatant was collected, centrifuged, aliquoted and stored at −20°C until LC-MS analysis.

For the LC-MS analysis, 500 µL cell aliquots were spiked with 38 µL internal standard mix and steroids subsequently extracted using solid-phase extraction on an OasisPrime HLB 96-well plate according to the protocol previously published (Andrieu *et al.* 2022). The LC-MS system consists of a Vanquish UHPLC (equipped with an ACQUITY UPLC HSS T3 Column, 100 Å, 1.8 µm, 1 mm × 100 mm; Waters, Switzerland) coupled to a Q Exactive Orbitrap Plus (both from ThermoFisher Scientific). Separation was achieved using gradient elution over 17 min using water and methanol (mobile phase B) both supplemented with 0.1 % formic acid (all Sigma-Aldrich) as mobile phases. The separation of steroid metabolites was achieved through the following elution gradient (at a constant flow of 0.15 mL/min): 0–0.5 min 1% B, 0.5–1 min linear gradient to 1–46% B, 1–4 min 46%, 4–12 min linear gradient 46–73% B, 12–12.5-min linear gradient 73–99% B, 12.5–14.5 min 99% B, 14.5–15-min linear gradient to 1% B, and 15–17 min 1% B. All LC-MS grade solvents required for analysis were from BioSolve (Switzerland).

Data analysis was performed using TraceFinder 4.1 (ThermoFisher Scientific).

Steroid hormone concentrations are displayed in nmol/L. The lower limit of accurate quantification (LLOQ) was 0.085 nmol/L for Aldo, 0.705 nmol/L for corticosterone, 0.476 nmol/L for progesterone, and 0.092 nmol/L for DOC. 18-OH-corticosterone was detected in the mass channel of corticosterone (m/z 347.2217), its elution time confirmed from timepoint 0h cell aliquots and it was quantified relative to the calibration curve of corticosterone.

For each batch of LC-MS analysis the same positive control H295R cells + AngII was used as internal control. The steroid hormone concentrations after 24 h were compared to the initial baseline steroid hormone concentrations at timepoint 0h. Assays were performed in triplicate, except for HAEC and HRMC cells. HAEC and HRMC assays were performed only once due to material limits.

### Statistical methods

Three independent cell culture experiments were performed per cell line, except for HAEC and HRMC. Due to a delivery bottleneck, the experiments with HAEC and HRMC cells were performed only once. PBMC experiments were done 6× with healthy subjects and 5× with PA patients.

Data in tables and figures are presented as mean ± s.e.m. An unpaired parametric *t*-test was used to compare two parameters with each other.

Significance was assigned at *P* < 0.05.

All statistical analyses were performed using GraphPad PRISM version 9.

## Results

### mRNA expression of CYP11B2

JEG-3, HTR-8/SV neo, BeWo, HUVEC, HUAEC, HAEC, HLEC, HRGEC, HRMC, HEK293, PBMCs, H295R and COS-7/CYP11B2 cells were cultured as described above. RNA was isolated and real-time PCR was performed to detect mRNA levels of *CYP11B2*. No expression of *CYP11B2* could be detected in JEG-3, HTR-8/SV neo, BeWo, HUVEC, HUAEC, HAEC, HLEC, HRGEC, HRMC, HEK293 cells and in PBMCs of healthy subjects and PA patients (ct values > 35, 50 cycles). In the positive control H295R cells, the baseline *CYP11B2* expression levels were ct ~34, and dropped upon Ang II stimulation to ct ~26 as expected. COS-7 cells overexpressing *CYP11B2* showed CYP11B2 ct values of ~15 independent of Ang II stimulation (Table 2).

**Table 2:** mRNA expression of CYP11B2 shown as ct values.

	ct CYP11B2 no Ang II	ct CYP11B2 + Ang II
JEG-3	>35	>35
HTR-8/SV neo	>35	>35
BeWo	>35	>35
HUVEC	>35	>35
HUAEC	>35	>35
HAEC	>35	>35
HLEC	>35	>35
HRGEC	>35	>35
HRMC	>35	>35
HEK293	>35	>35
PBMCs, healthy subjects	>35	>35
PBMCs, PA patients	>35	>35
H295R	34	26
COS-7 + CYP11B2 plasmid	15	15
COS-7 + empty plasmid	>35	>35

### mRNA expression of AGTR1 and AGTR2

JEG-3, HTR-8/SV neo, BeWo, HUVEC, HUAEC, HAEC, HLEC, HRGEC, HRMC, HEK293, H295R, and COS-7/CYP11B2 cells were cultured as described above. RNA was isolated and real-time PCR was performed to detect mRNA levels of *AGTR1* and *AGTR2.* Results are shown in Supplementary Table 5.

### Production of *de novo* steroid hormones from the substrates progesterone, DOC, corticosterone, and 18-OH-corticosterone

Supernatant from cell experiments were collected and steroid hormone production assessed with a high-resolution LC-MS-based method. Most results are shown in absolute values, nmol/L (mean ± s.e.m.), represented in tables. The concentration of each metabolite at timepoint 0h is compared to its concentration at time point 24 h. *P*-values are displayed directly next to the metabolites. NA, not assessed; ND, not detected.

**Figure 1:**
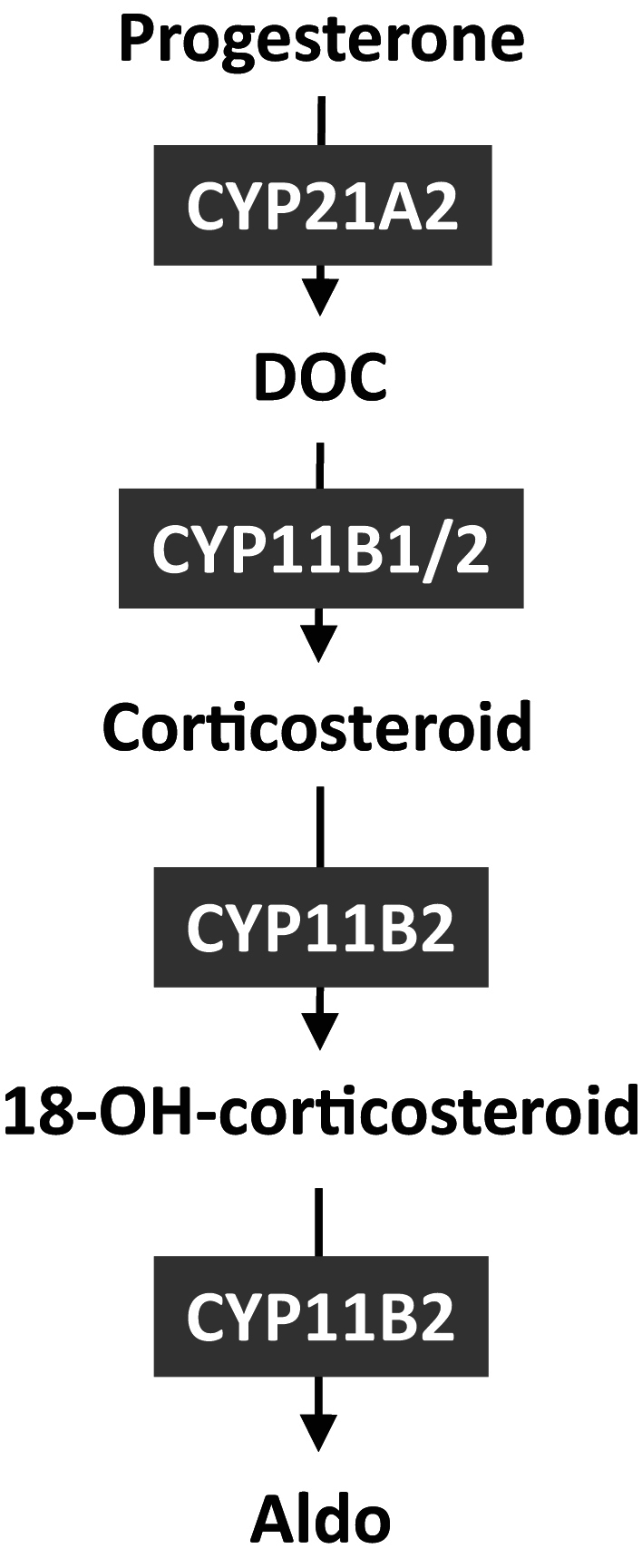
Aldo production pathway.

The Aldo production pathway is given in Fig. 1.

### Absolute values of steroid hormone metabolites in CYP11B2 transfected COS-7 cells supplemented with steroid hormone substrates DOC or corticosterone

**Figure 2:**
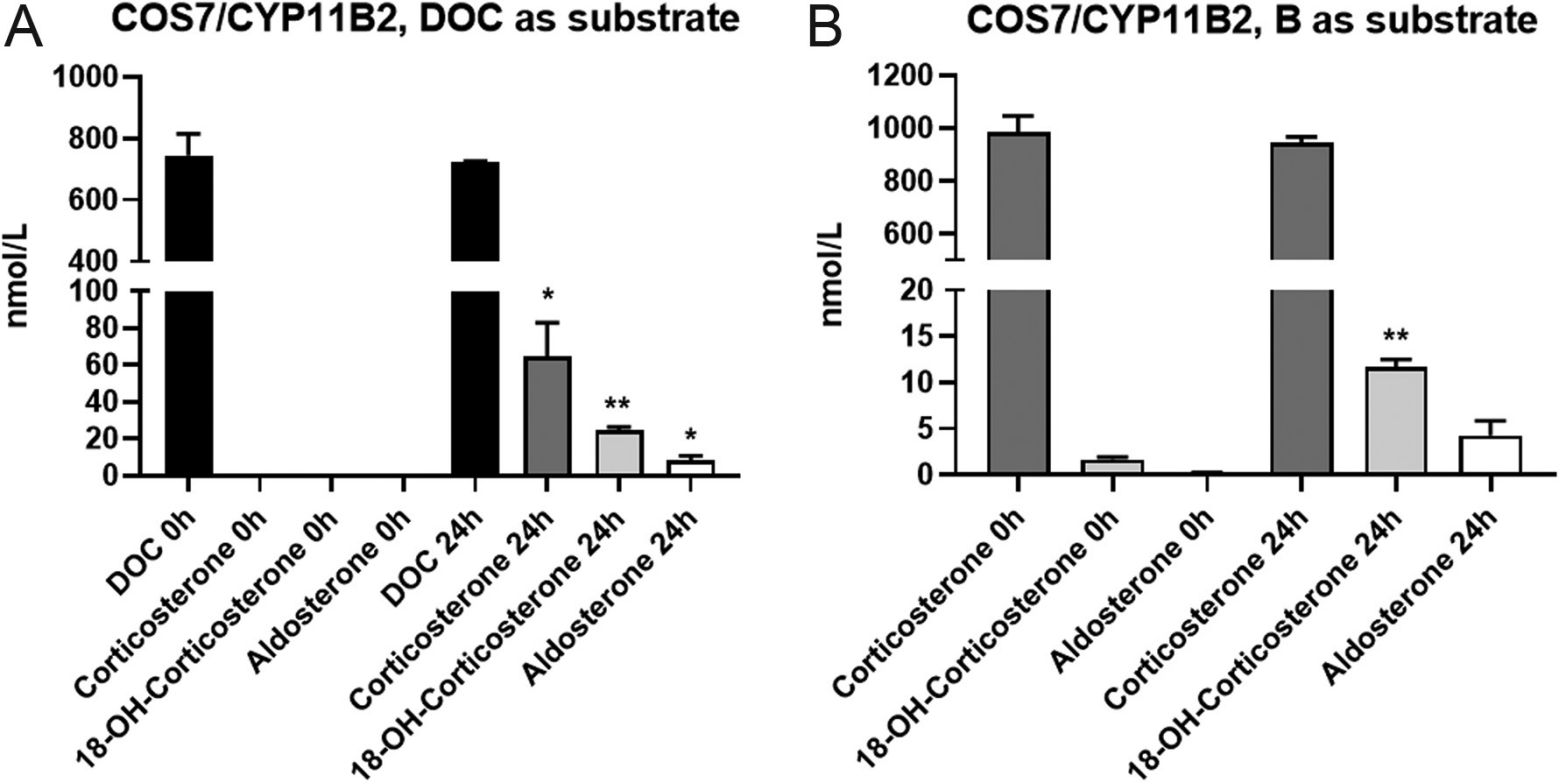
(A) Conversion of DOC to corticosterone, 18-OH-corticosterone, and Aldo in COS-7 cells transfected with CYP11B2. *t*-test; DOC 0 h vs DOC 24 h: ns, *P* = 0.713; corticosterone 0 h vs corticosterone 24 h: *P = 0.038; 18-OH-corticosterone 0 h vs 18-OH-corticosterone 24 h: **P = 0.002; Aldo 0 h vs Aldo 24 h: *P = 0.034. (B) Conversion of corticosterone to 18-OH-corticosterone and Aldo in COS-7 cells transfected with CYP11B2. *t*-test; corticosterone 0 h vs corticosterone 24 h: ns, *P* = 0.471; 18-OH-corticosterone 0 h vs 18-OH-corticosterone 24 h: **P = 0.004; Aldo 0 h vs Aldo 24 h: ns, *P* = 0.066. Time points 0 h and 24 h are shown. Steroid hormone data are displayed in nmol/L. *n* = 3, unpaired parametric *t*-test.

COS-7 cells overexpressing *CYP11B2* converted the substrate DOC to corticosterone, 18-OH-corticosterone, and Aldo, and these cells metabolized the substrate corticosterone to 18-OH-corticosterone and Aldo (Fig. 2).

### COS-7 cells

Absolute values of steroid hormone metabolites in COS-7 cells transfected with CYP11B2 and supplemented with the steroid hormone substrates progesterone, DOC, corticosterone, and 18-OH-corticosterone without and with Ang II (Table 3).

**Table 3:** Absolute values of steroid hormone metabolites (nmol/L) in CYP11B2 transfected COS-7 cells.

	Time	Mean ± s.e.m.	*P*	
**Progesterone as substrate**				
Progesterone	0 h	754.2 ± 135.1	0.313	ns
DOC	0 h	0.0 ± 0.0	0.269	ns
Corticosterone	0 h	0.0 ± 0.0	>0.999	ns
18-OH-corticosterone	0 h	0.0 ± 0.0	>0.999	ns
Aldosterone	0 h	0.0 ± 0.0	>0.999	ns
Progesterone	24 h	567.4 ± 35.0		
DOC	24 h	3.2 ± 2.1		
Corticosterone	24 h	0.0 ± 0.0		
18-OH-corticosterone	24 h	0.0 ± 0.0		
Aldosterone	24 h	0.0 ± 0.0		
**DOC as substrate**				
DOC	0 h	745.8 ± 49.8	0.713	ns
Corticosterone	0 h	0.0 ± 0.0	0.038	*
18-OH-corticosterone	0 h	0.0 ± 0.0	0.002	**
Aldosterone	0 h	0.0 ± 0.0	0.034	*
DOC	24 h	724.7 ± 1.4		
Corticosterone	24 h	64.6 ± 13.0		
18-OH-corticosterone	24 h	24.8 ± 1.1		
Aldosterone	24 h	8.4 ± 1.6		
**Corticosterone as substrate**				
Corticosterone	0 h	986.2 ± 42.8	0.471	ns
18-OH-corticosterone	0 h	1.7 ± 0.2	0.004	**
Aldosterone	0 h	0.1 ± 0.1	0.066	ns
Corticosterone	24 h	946.4 ± 14.4		
18-OH-corticosterone	24 h	11.6 ± 0.6		
Aldosterone	24 h	4.3 ± 1.1		
**18-OH-corticosterone as substrate**				
18-OH-corticosterone	0 h	1037.0 ± 179.5	0.651	ns
Aldosterone	0 h	0.5 ± 0.5	0.423	ns
18-OH-corticosterone	24 h	868.2 ± 265.5		
Aldosterone	24 h	0.0 ± 0.0		
**Progesterone + Ang II as substrate**				
Progesterone	0 h	642.3 ± 130.4	0.9759	ns
DOC	0 h	2.1 ± 2.1	0.7213	ns
Corticosterone	0 h	0.0 ± 0.0	>0.999	ns
18-OH-corticosterone	0 h	0.0 ± 0.0	>0.999	ns
Aldosterone	0 h	0.0 ± 0.0	>0.999	ns
Progesterone	24 h	637.8 ± 12.4		
DOC	24 h	3.9 ± 3.9		
Corticosterone	24 h	0.0 ± 0.0		
18-OH-corticosterone	24 h	0.0 ± 0.0		
Aldosterone	24 h	0.0 ± 0.0		
**DOC + Ang II as substrate**				
DOC	0 h	739.5 ± 90.3	0.635	ns
Corticosterone	0 h	0.0 ± 0.0	0.009	**
18-OH-corticosterone	0 h	0.0 ± 0.0	0.004	**
Aldosterone	0 h	0.0 ± 0.0	0.008	**
DOC	24 h	675.0 ± 73.4		
Corticosterone	24 h	60.6 ± 5.8		
18-OH-corticosterone	24 h	22.2 ± 1.4		
Aldosterone	24 h	7.6 ± 0.7		
**Corticosterone + Ang II as substrate**				
Corticosterone	0 h	962.8 ± 60.0	0.443	ns
18-OH-corticosterone	0 h	1.8 ± 0.0	0.995	ns
Aldosterone	0 h	0.0 ± 0.0	0.999	ns
Corticosterone	24 h	903.4 ± 23.4		
18-OH-corticosterone	24 h	14.8 ± 4.7		
Aldosterone	24 h	4.0 ± 0.9		
**18-OH-corticosterone + Ang II as substrate**				
18-OH-corticosterone	0 h	1068.0 ± 113.7	0.989	ns
Aldosterone	0 h	0.3 ± 0.3	>0.999	ns
18-OH-corticosterone	24 h	1001.0 ± 371.5		
Aldosterone	24 h	0.0 ± 0.0		

*P < 0.05, **P < 0.01, ***P< 0.0001; *P* > 0.05 not significant (ns).

### H295R cells

Absolute values of steroid hormone metabolites in H295R cells supplemented with the steroid hormone substrates progesterone, DOC, corticosterone, and 18-OH-corticosterone without and with Ang II (Table 4).

**Table 4:** Absolute values of steroid hormone metabolites (nmol/L) in H295R adrenal cells.

	Time	Mean ± s.e.m.	*P*	
**Progesterone as substrate**				
Progesterone	0 h	952.9 ± 23.5	<0.0001	***
DOC	0 h	0 ± 0	0.032	*
Corticosterone	0 h	0 ± 0	0.012	*
18-OH-corticosterone	0 h	NA		
Aldosterone	0 h	0 ± 0	0.062	ns
Progesterone	24 h	0.4 ± 0.2		
DOC	24 h	56.3 ± 17.4		
Corticosterone	24 h	4.5 ± 1.0		
18-OH-corticosterone	24 h	NA		
Aldosterone	24 h	0.4 ± 0.2		
**DOC as substrate**				
DOC	0 h	1133.0 ± 177.7	0.004	**
Corticosterone	0 h	0.3 ± 0.2	0.006	**
18-OH-corticosterone	0 h	NA		
Aldosterone	0 h	0 ± 0	0.08	ns
DOC	24 h	65.7 ± 18.0		
Corticosterone	24 h	6.6 ± 1.2		
18-OH-corticosterone	24 h	NA		
Aldosterone	24 h	0.6 ± 0.2		
**Corticosterone as substrate**				
Corticosterone	0 h	1594.0 ± 186.2	0.405	ns
18-OH-corticosterone	0 h	NA		
Aldosterone	0 h	0.0 ± 0.0	0.083	ns
Corticosterone	24 h	1377.0 ± 141.8		
18-OH-corticosterone	24 h	NA		
Aldosterone	24 h	0.6 ± 0.2		
**18-OH-corticosterone as substrate**				
18-OH-corticosterone	0 h	911.7 ± 69.9	0.342	ns
Aldosterone	0 h	0.5 ± 0.1	0.166	ns
18-OH-corticosterone	24 h	820.9 ± 47.0		
Aldosterone	24 h	0.8 ± 0.2		
**Progesterone + Ang II as substrate**				
Progesterone	0 h	910.6 ± 93.2	0.001	**
DOC	0 h	0 ± 0	0.022	*
Corticosterone	0 h	0 ± 0	0.009	**
18-OH-corticosterone	0 h	NA		
Aldosterone	0 h	0 ± 0	0.003	**
Progesterone	24 h	0.5 ± 0.1		
DOC	24 h	119.9 ± 32.9		
Corticosterone	24 h	43.0 ± 8.9		
18-OH-corticosterone	24 h	NA		
Aldosterone	24 h	2.0 ± 0.3		
**DOC + Ang II as substrate**				
DOC	0 h	1031.0 ± 114.2	0.002	**
Corticosterone	0 h	0.4 ± 0.3	0.003	**
18-OH-corticosterone	0 h	NA		
Aldosterone	0 h	0 ± 0	< 0.0001	***
DOC	24 h	139.3 ± 28.4		
Corticosterone	24 h	51.3 ± 8.1		
18-OH-corticosterone	24 h	NA		
Aldosterone	24 h	2.1 ± 0.7		
**Corticosterone + Ang II as substrate**				
Corticosterone	0 h	1746.0 ± 295.2	0.263	ns
18-OH-corticosterone	0 h	NA		
Aldosterone	0 h	0.0 ± 0.0	0.0001	**
Corticosterone	24 h	1324.0 ± 136.2		
18-OH-corticosterone	24 h	NA		
Aldosterone	24 h	2.1 ± 0.7		
**18-OH-corticosterone + Ang II as substrate**				
18-OH-corticosterone	0 h	888.8 ± 24.3	0.333	ns
Aldosterone	0 h	0.3 ± 0.2	0.008	**
18-OH-corticosterone	24 h	792.2 ± 84.4		
Aldosterone	24 h	1.1 ± 0.0		

*P < 0.05, **P < 0.01, ***P < 0.0001; *P* > 0.05 not significant (ns); NA, not assessed.

### Placental cell lines

Absolute values of steroid hormone metabolites in JEG-3, BeWo, and HTR-8/SVneo cells supplemented with the steroid hormone substrates progesterone, DOC, corticosterone, and 18-OH-corticosterone without and with Ang II (Table 5).

**Table 5:** Absolute values of steroid hormone metabolites in JEG-3, BeWo, and HTR-8/Svneo placental cell lines.

	Time	JEG-3 (nmol/L)			BeWo (nmol/L)			HTR-8/SVneo (nmol/L)		
		Mean ± s.e.m.	*P*		Mean ± s.e.m.	*P*		Mean ± s.e.m.	*P*	
**Progesterone as substrate**										
Progesterone	0 h	1339.0 ± 132.6	0.189	ns	803.4 ± 57.1	0.236	ns	1123.0 ± 144.9	0.061	ns
DOC	0 h	0.0 ± 0.0	0.177	ns	0.0 ± 0.0	0.012	*	0.0 ± 0.0	>0.999	ns
Corticosterone	0 h	0.0 ± 0.0	>0.999	ns	0.0 ± 0.0	>0.999	ns	0.0 ± 0.0	>0.999	ns
18-OH-corticosterone	0 h	0.0 ± 0.0	>0.999	ns	0.0 ± 0.0	>0.999	ns	0.0 ± 0.0	>0.999	ns
Aldosterone	0 h	0.0 ± 0.0	>0.999	ns	0.0 ± 0.0	>0.999	ns	0.0 ± 0.0	>0.999	ns
Progesterone	24 h	1090.0 ± 85.70			540.9 ± 179.7			724.2 ± 51.5		
DOC	24 h	0.2 ± 0.1			0.1 ± 0.0			0.0 ± 0.0		
Corticosterone	24 h	0.0 ± 0.0			0.0 ± 0.0			0.0 ± 0.0		
18-OH-corticosterone	24 h	0.0 ± 0.0			0.0 ± 0.0			0.0 ± 0.0		
Aldosterone	24 h	0.0 ± 0.0			0.0 ± 0.0			0.0 ± 0.0		
**DOC as substrate**										
DOC	0 h	1460.0 ± 163.0	0.029	*	792.2 ± 75.0	0.228	ns	1304.0 ± 245.5	0.131	ns
Corticosterone	0 h	0.0 ± 0.0	>0.999	ns	0.0 ± 0.0	>0.999	ns	0.2 ± 0.4	0.937	ns
18-OH-corticosterone	0 h	0.0 ± 0.0	>0.999	ns	0.0 ± 0.0	>0.999	ns	0.0 ± 0.0	>0.999	ns
Aldosterone	0 h	0.0 ± 0.0	>0.999	ns	0.0 ± 0.0	>0.999	ns	0.0 ± 0.0	>0.999	ns
DOC	24 h	1141.0 ± 24.5			639.0 ± 77.3			1001.0 ± 129.9		
Corticosterone	24 h	0.0 ± 0.0			0.0 ± 0.0			0.2 ± 0.3		
18-OH-corticosterone	24 h	0.0 ± 0.0			0.0 ± 0.0			0.0 ± 0.0		
Aldosterone	24 h	0.0 ± 0.0			0.0 ± 0.0			0.0 ± 0.0		
**Corticosterone as substrate**										
Corticosterone	0 h	1532.0 ± 443.0	0.874	ns	1076.0 ± 90.4	0.959	ns	1727.0 ± 204.2	0.18	ns
18-OH-corticosterone	0 h	0.0 ± 0.0	>0.999	ns	2.1 ± 0.3	0.664	ns	0.0 ± 0.0	>0.999	ns
Aldosterone	0 h	0.1 ± 0.0	>0.999	ns	0.0 ± 0.0	>0.999	ns	0.0 ± 0.0	>0.999	ns
Corticosterone	24 h	1483.0 ± 246.3			1065.0 ± 162.4			1536.0 ± 34.7		
18-OH-corticosterone	24 h	0.0 ± 0.0			1.9 ± 0.1			0.0 ± 0.0		
Aldosterone	24 h	0.0 ± 0.0			0.0 ± 0.0			0.0 ± 0.0		
**18-OH-corticosterone as substrate**										
18-OH-corticosterone	0 h	1285.0 ± 253.3	0.241	ns	827.9 ± 162.5	0.613	ns	966.2 ± 207.9	0.540	ns
Aldosterone	0 h	0.5 ± 0.1	0.107	ns	0.2 ± 0.2	0.901	ns	0.5 ± 0.2	0.600	ns
18-OH-corticosterone	24 h	1047.0 ± 160.3			707.7 ± 147.6			851.8 ± 211.6		
Aldosterone	24 h	0.4 ± 0.1			0.2 ± 0.1			0.4 ± 0.2		
**Progesterone + Ang II as substrate**										
Progesterone	0 h	1252.0 ± 120.7	0.111	ns	709.7 ± 105.8	0.512	ns	1227.0 ± 122.2	0.0373	*
DOC	0 h	0.0 ± 0.0	0.374	ns	0.0 ± 0.0	0.016	*	0.0 ± 0.0	>0.999	ns
Corticosterone	0 h	0.0 ± 0.0	>0.999	ns	0.0 ± 0.0	>0.999	ns	0.0 ± 0.0	>0.999	ns
18-OH-corticosterone	0 h	0.0 ± 0.0	>0.999	ns	0.0 ± 0.0	>0.999	ns	0.0 ± 0.0	>0.999	ns
Aldosterone	0 h	0.0 ± 0.0	>0.999	ns	0.0 ± 0.0	>0.999	ns	0.0 ± 0.0	>0.999	ns
Progesterone	24 h	943.3 ± 91.7			563.8 ± 172.9			762.1 ± 89.5		
DOC	24 h	0.1 ± 0.1			0.1 ± 0.0			0.0 ± 0.0		
Corticosterone	24 h	0.0 ± 0.0			0.0 ± 0.0			0.0 ± 0.0		
18-OH-corticosterone	24 h	0.0 ± 0.0			0.0 ± 0.0			0.0 ± 0.0		
Aldosterone	24 h	0.0 ± 0.0			0.0 ± 0.0			0.0 ± 0.0		
**DOC + Ang II as substrate**										
DOC	0 h	1375.0 ± 249.1	0.258	ns	923.1 ± 49.6	0.157	ns	1364.0 ± 176.8	0.116	ns
Corticosterone	0 h	0.0 ± 0.0	>0.999	ns	0.0 ± 0.0	>0.999	ns	0.3 ± 0.4	0.447	ns
18-OH-corticosterone	0 h	0.0 ± 0.0	>0.999	ns	0.0 ± 0.0	>0.999	ns	0.0 ± 0.0	>0.999	ns
Aldosterone	0 h	0.0 ± 0.0	>0.999	ns	0.0 ± 0.0	>0.999	ns	0.0 ± 0.0	>0.999	ns
DOC	24 h	1144.0 ± 173.1			598.3 ± 180.3			1050.0 ± 207.4		
Corticosterone	24 h	0.0 ± 0.0			0.0 ± 0.0			0.1 ± 0.1		
18-OH-corticosterone	24 h	0.0 ± 0.0			0.0 ± 0.0			0.0 ± 0.0		
Aldosterone	24 h	0.0 ± 0.0			0.0 ± 0.0			0.0 ± 0.0		
**Corticosterone + Ang II as substrate**										
Corticosterone	0 h	1778.0 ± 244.3	0.095	ns	955.2 ± 97.2	0.211	ns	1595.0 ± 270.8	0.870	ns
18-OH-corticosterone	0 h	0.0 ± 0.0	>0.999	ns	1.7 ± 0.1	0.413	ns	0.0 ± 0.0	>0.9999	ns
Aldosterone	0 h	0.1 ± 0.1	0.618	ns	0.0 ± 0.0	>0.999	ns	0.1 ± 0.1	>0.9999	ns
Corticosterone	24 h	1384.0 ± 195.9			777.3 ± 69.6			1656.0 ± 545.1		
18-OH-corticosterone	24 h	0.0 ± 0.0			1.4 ± 0.3			0.0 ± 0.0		
Aldosterone	24 h	0.0 ± 0.0			0.0 ± 0.0			0.0 ± 0.0		
**18-OH-corticosterone + Ang II as substrate**										
18-OH-corticosterone	0 h	1197.0 ± 132.0	0.043	*	712.1 ± 118.9	0.338	ns	927.7 ± 274.0	0.394	ns
Aldosterone	0 h	0.5 ± 0.0	0.104	ns	0.2 ± 0.2	0.694	ns	0.5 ± 0.1	0.917	ns
18-OH-corticosterone	24 h	944.1 ± 70.5			571.3 ± 51.4			756.3 ± 146.7		
Aldosterone	24 h	0.4 ± 0.1			0.3 ± 0.2			0.5 ± 0.2		

*P < 0.05, **P < 0.01, ***P < 0.0001; *P* > 0.05 not significant (ns).

### Endothelial cell lines

Absolute values of steroid hormone metabolites in HUVEC, HUAEC, HAEC, HRGEC, and HLEC cells supplemented with the steroid hormone substrates progesterone, DOC, corticosterone, and 18-OH-corticosterone withou t and with Ang II (Table 6).

**Table 6:** Absolute values of steroid hormone metabolites in HUVEC, HUAEC, HAEC, HRGEC, and HLEC endothelial cell lines.

	Time	HUVEC (nmol/L)			HUAEC (nmol/L)			HAEC (nmol/L)		HRGEC (nmol/L)			HLEC (nmol/L)		
		Mean ± s.e.m.	*P*		Mean ± s.e.m.	*P*		Mean ± s.e.m.	*P*	Mean ± s.e.m.	*P*		Mean ± s.e.m.	*P*	
**Progesterone as substrate**															
Progesterone	0 h	680.4 ± 54.7	0.656	ns	671.9 ± 42.6	0.843	ns	1100		864.0 ± 109.9	0.133	ns	850.1 ± 53.1	0.163	ns
DOC	0 h	0.0 ± 0.0	>0.999	ns	0.6 ± 0.5	0.481	ns	0.0		2.6 ± 2.6	0.916	ns	4.1 ± 4.1	0.981	ns
Corticosterone	0 h	0.0 ± 0.0	>0.999	ns	0.0 ± 0.0	<0.0001	***	0.0		0.0 ± 0.0	0.116	ns	0.0 ± 0.0	>0.999	ns
18-OH-corticosterone	0 h	0.0 ± 0.0	>0.999	ns	0.0 ± 0.0	>0.999	ns	0.0		0.0 ± 0.0	>0.999	ns	0.0 ± 0.0	>0.999	ns
Aldosterone	0 h	0.0 ± 0.0	>0.999	ns	0.0 ± 0.0	>0.999	ns	0.0		0.0 ± 0.0	>0.999	ns	0.0 ± 0.0	>0.999	ns
Progesterone	24 h	644.6 ± 50.5			695.2 ± 101.6			910.8		646.7 ± 35.3			1137.0 ± 159.5		
DOC	24 h	0.0 ± 0.0			0.2 ± 0.1			0.1		2.2 ± 2.2			4.3 ± 4.2		
Corticosterone	24 h	0.0 ± 0.0			4.8 ± 0.2			6.2		2.4 ± 1.2			0.0 ± 0.0		
18-OH-corticosterone	24 h	0.0 ± 0.0			0.0 ± 0.0			0.0		0.0 ± 0.0			0.0 ± 0.0		
Aldosterone	24 h	0.0 ± 0.0			0.0 ± 0.0			0.0		0.0 ± 0.0			0.0 ± 0.0		
**DOC as substrate**															
DOC	0 h	1028.0 ± 75.3	0.627	ns	824.0 ± 60.3	0.803	ns	1108		737.2 ± 118.0	0.347	ns	992.5 ± 247.1	0.780	ns
Corticosterone	0 h	0.0 ± 0.0	>0.999	ns	0.0 ± 0.0	0.169	ns	2.4		0.0 ± 0.0	<0.0001	***	0.1 ± 0.1	0.374	ns
18-OH-corticosterone	0 h	0.0 ± 0.0	>0.999	ns	0.0 ± 0.0	>0.999	ns	0.0		0.0 ± 0.0	>0.999	ns	0.0 ± 0.0	0.120	ns
Aldosterone	0 h	0.0 ± 0.0	>0.999	ns	0.1 ± 0.1	0.374	ns	0.0		0.1 ± 0.1	>0.999	ns	0.0 ± 0.0	>0.999	ns
DOC	24 h	905.8 ± 219.0			889.8 ± 238.8			992.3		561.0 ± 115.8			1079.0 ± 149.8		
Corticosterone	24 h	0.0 ± 0.0			11.9 ± 7.1			5.6		3.5 ± 0.0			0.0 ± 0.0		
18-OH-corticosterone	24 h	0.0 ± 0.0			0.0 ± 0.0			0.0		0.0 ± 0.0			1.2 ± 0.6		
Aldosterone	24 h	0.0 ± 0.0			0.0 ± 0.0			0.0		0.0 ± 0.0			0.0 ± 0.0		
Corticosterone as substrate															
Corticosterone	0 h	903.6 ± 49.6	0.331	ns	760.5 ± 103.4	0.324	ns	1197.6		1171.0 ± 137.2	0.850	ns	1405.0 ± 496.9	0.786	ns
18-OH-corticosterone	0 h	2.0 ± 0.2	>0.999	ns	0.0 ± 0.0	>0.999	ns	0.0		0.0 ± 0.0	>0.999	ns	2.5 ± 1.3	0.108	ns
Aldosterone	0 h	0.0 ± 0.0	>0.999	ns	0.0 ± 0.0	>0.999	ns	0.0		0.0 ± 0.0	>0.999	ns	0.0 ± 0.0	>0.999	ns
Corticosterone	24 h	996.4 ± 67.9			936.8 ± 118.1			1220.6		1129.0 ± 153.7			1586.0 ± 371.3		
18-OH-corticosterone	24 h	1.8 ± 0.1			0.0 ± 0.0			0.0		0.0 ± 0.0			5.6 ± 0.8		
Aldosterone	24 h	0.0 ± 0.0			0.0 ± 0.0			0.0		0.0 ± 0.0			0.0 ± 0.0		
**18-OH-corticosterone as substrate**															
18-OH-corticosterone	0 h	663.9 ± 128.1	0.719	ns	1005.0 ± 102.7	0.238	ns	968.6		1152.0 ± 58.3	0.101	ns	1214.0 ± 423.1	0.780	ns
Aldosterone	0 h	0.3 ± 0.1	0.284	ns	0.4 ± 0.1	0.733	ns	0.6		0.3 ± 0.1	0.797	ns	0.6 ± 0.2	0.666	ns
18-OH-corticosterone	24 h	566.3 ± 90.5			1313.0 ± 197.0			1063.5		1287.0 ± 24.4			1026.0 ± 465.1		
Aldosterone	24 h	0.4 ± 0.1			0.3 ± 0.0			0.9		0.3 ± 0.0			0.7 ± 0.2		
**Progesterone + Ang II as substrate**															
Progesterone	0 h	856.3 ± 91.2	0.135	ns	613.2 ± 47.9	0.907	ns	902.7		890.7 ± 139.4	0.876	ns	688.0 ± 23.5	0.049	*
DOC	0 h	0.0 ± 0.0	>0.999	ns	0.0 ± 0.0	0.049	*	0.0		0.0 ± 0.0	0.43	ns	0.0 ± 0.0	>0.999	ns
Corticosterone	0 h	0.0 ± 0.0	>0.999	ns	0.0 ± 0.0	0.042	*	0.0		0.0 ± 0.0	<0.0001	***	0.0 ± 0.0	>0.999	ns
18-OH-corticosterone	0 h	0.0 ± 0.0	>0.999	ns	0.0 ± 0.0	>0.999	ns	0.0		0.0 ± 0.0	>0.999	ns	0.0 ± 0.0	>0.999	ns
Aldosterone	0 h	0.0 ± 0.0	>0.999	ns	0.1 ± 0.1	0.374	ns	0.0		0.0 ± 0.0	>0.999	ns	0.0 ± 0.0	>0.999	ns
Progesterone	24 h	673.8 ± 34.9			604.4 ± 51.9			1036.4		856.9 ± 148.2			894.0 ± 70.1		
DOC	24 h	0.0 ± 0.0			0.1 ± 0.0			0.2		0.1 ± 0.0			0.0 ± 0.0		
Corticosterone	24 h	0.0 ± 0.0			7.7 ± 2.6			3.4		3.3 ± 0.1			0.0 ± 0.0		
18-OH-corticosterone	24 h	0.0 ± 0.0			0.0 ± 0.0			0.0		0.0 ± 0.0			0.0 ± 0.0		
Aldosterone	24 h	0.0 ± 0.0			0.0 ± 0.0			0.0		0.0 ± 0.0			0.0 ± 0.0		
DOC + Ang II as substrate															
DOC	0 h	1098.0 ± 169.5	0.132	ns	872.4 ± 179.8	0.779	ns	940.5		930.2 ± 177.9	0.838	ns	1289.0 ± 520.9	0.827	ns
Corticosterone	0 h	0.0 ± 0.0	>0.999	ns	0.0 ± 0.0	0.0003	**	2.5		0.0 ± 0.0	0.0003	**	0.0 ± 0.0	0.374	ns
18-OH-corticosterone	0 h	0.0 ± 0.0	>0.999	ns	0.0 ± 0.0	>0.999	ns	0.0		0.0 ± 0.0	>0.999	ns	1.5 ± 1.5	0.529	ns
Aldosterone	0 h	0.0 ± 0.0	>0.999	ns	0.0 ± 0.0	>0.999	ns	0.0		0.0 ± 0.0	>0.999	ns	0.0 ± 0.0	>0.999	ns
DOC	24 h	770.7 ± 37.5			811.4 ± 94.3			1026.9		874.0 ± 186.5			1461.0 ± 520.0		
Corticosterone	24 h	0.0 ± 0.0			5.1 ± 0.4			3.6		3.4 ± 0.3			0.3 ± 0.3		
18-OH-corticosterone	24 h	0.0 ± 0.0			0.0 ± 0.0			0.0		0.0 ± 0.0			2.8 ± 1.3		
Aldosterone	24 h	0.0 ± 0.0			0.0 ± 0.0			0.0		0.0 ± 0.0			0.0 ± 0.0		
**Corticosterone + Ang II as substrate**															
Corticosterone	0 h	1020.0 ± 155.7	0.727	ns	1009.0 ± 121.1	0.765	ns	1179.4		980.2 ± 84.3	0.661	ns	897.8 ± 136.4	0.046	*
18-OH-corticosterone	0 h	2.1 ± 0.3	0.383	ns	0.0 ± 0.0	>0.999	ns	0.0		0.0 ± 0.0	>0.999	ns	1.7 ± 0.7	0.139	ns
Aldosterone	0 h	0.0 ± 0.0	>0.999	ns	0.1 ± 0.1	0.374	ns	0.0		0.0 ± 0.0	>0.999	ns	0.0 ± 0.0	>0.999	ns
Corticosterone	24 h	961.6 ± 15.5			948.8 ± 146.2			1126.9		937.0 ± 35.3			1718.0 ± 251.8		
18-OH-corticosterone	24 h	1.7 ± 0.2			0.0 ± 0.0			0.0		0.0 ± 0.0			8.4 ± 3.5		
Aldosterone	24 h	0.0 ± 0.0			0.0 ± 0.0			0.0		0.0 ± 0.0			0.0 ± 0.0		
**18-OH-corticosterone + Ang II as substrate**															
18-OH-corticosterone	0 h	881.3 ± 44.8	0.135	ns	1069.0 ± 132.4	0.177	ns	982.1		1140.0 ± 67.9	0.360	ns	573.9 ± 104.1	0.116	ns
Aldosterone	0 h	0.4 ± 0.1	0.662	ns	0.3 ± 0.1	0.942	ns	0.7		0.3 ± 0.1	0.941	ns	0.3 ± 0.0	0.007	**
18-OH-corticosterone	24 h	671.0 ± 103.3			1377.0 ± 52.4			884.5		1241.0 ± 70.7			803.6 ± 48.2		
Aldosterone	24 h	0.3 ± 0.1			0.3 ± 0.1			0.9		0.3 ± 0.1			0.6 ± 0.0		

*P < 0.05, **P < 0.01, ***P < 0.0001; *P* > 0.05 not significant (ns).

### Renal cell lines

Absolute values of steroid hormone metabolites in HRMC and HEK293 cells supplemented with the steroid hormone substrates progesterone, DOC, corticosterone, and 18-OH-corticosterone without and with Ang II (Table 7).

**Table 7:** Absolute values of steroid hormone metabolites in HRMC and HEK293 renal cell lines.

	Time	**HRMC (nmol/L)**		**HEK293 (nmol/L)**		
		Mean ± s.e.m.	*P*	Mean ± s.e.m.	*P*	
**Progesterone as substrate**						
Progesterone	0 h	1076.0		1101.0 ± 241.7	0.421	ns
DOC	0 h	0.0		0.0 ± 0.0	0.213	ns
Corticosterone	0 h	0.0		0.0 ± 0.0	>0.999	ns
18-OH-corticosterone	0 h	0.0		0.0 ± 0.0	>0.999	ns
Aldosterone	0 h	0.0		0.0 ± 0.0	>0.999	ns
Progesterone	24 h	1008.0		799.8 ± 232.7		
DOC	24 h	0.2		0.1 ± 0.0		
Corticosterone	24 h	5.2		0.0 ± 0.0		
18-OH-corticosterone	24 h	0.00		0.0 ± 0.0		
Aldosterone	24 h	0.00		0.0 ± 0.0		
**DOC as substrate**						
DOC	0 h	1188.0		1237.0 ± 255.4	0.132	ns
Corticosterone	0 h	0.0		0.0 ± 0.0	0.163	ns
18-OH-corticosterone	0 h	0.0		0.2 ± 0.2	0.400	ns
Aldosterone	0 h	0.0		0.0 ± 0.0	>0.999	ns
DOC	24 h	860.0		687.8 ± 137.5		
Corticosterone	24 h	4.8		0.5 ± 0.3		
18-OH-corticosterone	24 h	0.0		0.0 ± 0.0		
Aldosterone	24 h	0.0		0.0 ± 0.0		
**Corticosterone as substrate**						
Corticosterone	0 h	1267.7		2093.0 ± 343.1	0.469	ns
18-OH-corticosterone	0 h	0.0		2.8 ± 0.1	0.236	ns
Aldosterone	0 h	0.0		0.0 ± 0.0	>0.999	ns
Corticosterone	24 h	1411.3		1738.0 ± 282.8		
18-OH-corticosterone	24 h	0.0		2.6 ± 0.1		
Aldosterone	24 h	0.0		0.0 ± 0.0		
**18-OH-corticosterone as substrate**						
18-OH-corticosterone	0 h	796.4		901.9 ± 59.9	0.025	*
Aldosterone	0 h	0.2		0.4 ± 0.1	0.903	ns
18-OH-corticosterone	24 h	839.5		606.9 ± 58.9		
Aldosterone	24 h	0.4		0.4 ± 0.2		
**Progesterone + Ang II as substrate**						
Progesterone	0 h	968.0		1367.0 ± 220.8	0.309	ns
DOC	0 h	0.0		0.0 ± 0.0	0.258	ns
Corticosterone	0 h	0.0		0.0 ± 0.0	>0.999	ns
18-OH-corticosterone	0 h	0.0		0.0 ± 0.0	>0.999	ns
Aldosterone	0 h	0.0		0.0 ± 0.0	>0.999	ns
Progesterone	24 h	676.0		1011.0 ± 210.9		
DOC	24 h	0.2		0.1 ± 0.1		
Corticosterone	24 h	5.1		0.0 ± 0.0		
18-OH-corticosterone	24 h	0.0		0.0 ± 0.0		
Aldosterone	24 h	0.0		0.0 ± 0.0		
**DOC + Ang II as substrate**						
DOC	0 h	1192.0		1193.0 ± 229.2	0.400	ns
Corticosterone	0 h	0.0		0.2 ± 0.2	0.801	ns
18-OH-corticosterone	0 h	0.0		2.4 ± 2.4	0.376	ns
Aldosterone	0 h	0.0		0.0 ± 0.0	>0.999	ns
DOC	24 h	852.0		945.6 ± 129.0		
Corticosterone	24 h	4.6		0.1 ± 0.1		
18-OH-corticosterone	24 h	0.0		0.0 ± 0.0		
Aldosterone	24 h	0.0		0.0 ± 0.0		
**Corticosterone + Ang II as substrate**						
Corticosterone	0 h	1430.8		1535.0 ± 448.9	0.861	ns
18-OH-corticosterone	0 h	0.0		2.2 ± 0.3	0.854	ns
Aldosterone	0 h	0.0		0.0 ± 0.0	>0.999	ns
Corticosterone	24 h	1170.3		1688.0 ± 687.9		
18-OH-corticosterone	24 h	0.0		2.2 ± 0.4		
Aldosterone	24 h	0.0		0.0 ± 0.0		
**18-OH-corticosterone + Ang II as substrate**						
18-OH-corticosterone	0 h	808.7		853.1 ± 81.3	0.188	ns
Aldosterone	0 h	0.4		0.4 ± 0.3	0.368	ns
18-OH-corticosterone	24 h	651.9		699.6 ± 52.4		
Aldosterone	24 h	0.2		0.1 ± 0.1		

*P < 0.05, **P < 0.01, ***P < 0.0001; *P* > 0.05 not significant (ns).

### Peripheral blood mononuclear cells

Absolute values of steroid hormone metabolites in PBMCs of healthy subjects and of PA patients supplemented with the substrates progesterone, DOC, corticosterone, and 18-OH-corticosterone without and with Ang II (Table 8). Data of PBMCs are additionally shown as dot plots in Supplementary Figs. 3, 4, 5, and 6.

**Table 8:** Absolute values of steroid hormone metabolites in PBMCs from healthy subjects and PA patients.

	Time	**PBMC no PA (nmol/L)**			**PBMC with PA (nmol/L)**		
		Mean ± s.e.m.	*P*		Mean ± s.e.m.	*P*	
**Progesterone as substrate**							
Progesterone	0 h	924.7 ± 58.2	0.044	*	1072.0 ± 98.5	0.533	ns
DOC	0 h	0.0 ± 0.0	>0.999	ns	0.0 ± 0.0	0.676	ns
Corticosterone	0 h	0.0 ± 0.0	>0.999	ns	0.0 ± 0.0	>0.999	ns
18-OH-corticosterone	0 h	0.0 ± 0.0	>0.999	ns	0.0 ± 0.0	>0.999	ns
Aldosterone	0 h	0.0 ± 0.0	>0.999	ns	0.0 ± 0.0	>0.999	ns
Progesterone	24 h	721.1 ± 66.7			995.0 ± 65.0		
DOC	24 h	0.0 ± 0.0			0.1 ± 0.1		
Corticosterone	24 h	0.0 ± 0.0			0.0 ± 0.0		
18-OH-corticosterone	24 h	0.0 ± 0.0			0.0 ± 0.0		
Aldosterone	24 h	0.0 ± 0.0			0.0 ± 0.0		
**DOC as substrate**							
DOC	0 h	748.3 ± 166.7	0.744	ns	1010.0 ± 61.6	0.414	ns
Corticosterone	0 h	0.0 ± 0.0	>0.999	ns	0.0 ± 0.0	>0.999	ns
18-OH-corticosterone	0 h	0.0 ± 0.0	>0.999	ns	0.0 ± 0.0	>0.999	ns
Aldosterone	0 h	0.4 ± 0.4	0.911	ns	0.0 ± 0.0	>0.999	ns
DOC	24 h	715.2 ± 174.8			932.3 ± 66.2		
Corticosterone	24 h	0.0 ± 0.0			0.0 ± 0.0		
18-OH-corticosterone	24 h	0.0 ± 0.0			0.0 ± 0.0		
Aldosterone	24 h	0.4 ± 0.4			0.0 ± 0.0		
**Corticosterone as substrate**							
Corticosterone	0 h	813.5 ± 86.4	0.629	ns	1077.0 ± 52.5	0.732	ns
18-OH-corticosterone	0 h	0.0 ± 0.0	>0.999	ns	1.8 ± 0.5	>0.999	ns
Aldosterone	0 h	0.0 ± 0.0	>0.999	ns	0.0 ± 0.0	>0.999	ns
Corticosterone	24 h	859.2 ± 207.5			1105.0 ± 60.9		
18-OH-corticosterone	24 h	0.0 ± 0.0			1.9 ± 0.5		
Aldosterone	24 h	0.0 ± 0.0			0.0 ± 0.0		
**18-OH-corticosterone as substrate**							
18-OH-corticosterone	0 h	798.0 ± 63.2	0.075	ns	864.8 ± 60.5	0.573	ns
Aldosterone	0 h	0.4 ± 0.0	0.266	ns	0.1 ± 0.1	0.932	ns
18-OH-corticosterone	24 h	623.2 ± 61.2			810.6 ± 69.6		
Aldosterone	24 h	0.4 ± 0.0			0.1 ± 0.1		
**Progesterone + Ang II as substrate**							
Progesterone	0 h	986.9 ± 62.2	0.018	*	1019.0 ± 101.9	0.182	ns
DOC	0 h	0.0 ± 0.0	>0.999	ns	0.3 ± 0.2	0.141	ns
Corticosterone	0 h	0.0 ± 0.0	>0.999	ns	0.0 ± 0.0	>0.999	ns
18-OH-corticosterone	0 h	0.0 ± 0.0	>0.999	ns	0.0 ± 0.0	>0.999	ns
Aldosterone	0 h	0.0 ± 0.0	>0.999	ns	0.0 ± 0.0	>0.999	ns
Progesterone	24 h	754.2 ± 53.8			866.7 ± 21.2		
DOC	24 h	0.0 ± 0.0			0.0 ± 0.0		
Corticosterone	24 h	0.0 ± 0.0			0.0 ± 0.0		
18-OH-corticosterone	24 h	0.0 ± 0.0			0.0 ± 0.0		
Aldosterone	24 h	0.0 ± 0.0			0.0 ± 0.0		
**DOC + Ang II as substrate**							
DOC	0 h	882.9 ± 67.1	0.017	*	967.5 ± 75.6	0.748	ns
Corticosterone	0 h	0.0 ± 0.0	>0.999	ns	0.0 ± 0.0	>0.999	ns
18-OH-corticosterone	0 h	0.0 ± 0.0	>0.999	ns	0.0 ± 0.0	>0.999	ns
Aldosterone	0 h	0.0 ± 0.0	>0.999	ns	0.0 ± 0.0	>0.999	ns
DOC	24 h	664.0 ± 175.0			936.2 ± 55.9		
Corticosterone	24 h	0.0 ± 0.0			0.0 ± 0.0		
18-OH-corticosterone	24 h	0.0 ± 0.0			0.0 ± 0.0		
Aldosterone	24 h	0.0 ± 0.0			0.0 ± 0.0		
**Corticosterone + Ang II as substrate**							
Corticosterone	0 h	833.3 ± 305.0	0.511	ns	1128.0 ± 34.0	0.537	ns
18-OH-corticosterone	0 h	0.0 ± 0.0	>0.999	ns	2.2 ± 0.1	0.738	ns
Aldosterone	0 h	0.0 ± 0.0	>0.999	ns	0.0 ± 0.0	>0.999	ns
Corticosterone	24 h	934.8 ± 199.9			1177.0 ± 66.9		
18-OH-corticosterone	24 h	0.0 ± 0.0			2.3 ± 0.1		
Aldosterone	24 h	0.0 ± 0.0			0.0 ± 0.0		
**18-OH-corticosterone + Ang II as substrate**							
18-OH-corticosterone	0 h	774.6 ± 34.1	0.001	**	868.8 ± 60.3	0.757	ns
Aldosterone	0 h	0.4 ± 0.0	0.007	**	0.1 ± 0.1	0.908	ns
18-OH-corticosterone	24 h	515.5 ± 40.7			898.3 ± 69.6		
Aldosterone	24 h	0.3 ± 0.0			0.1 ± 0.1		

*P < 0.05, **P < 0.01, ***P < 0.0001; *P* > 0.05 not significant (ns).

### Aldosterone production in all assessed cell lines and primary cells

Aldo production in COS-7 cells transfected with CYP11B2 and supplemented with the substrate progesterone or 18-OH-corticosterone was 0.0 ± 0.0 nmol/L after 24h no matter if stimulated with Ang II or not. With DOC as substrate, Aldo production was 8.4 ± 1.6 nmol/L (no AngII) and 7.6 ± 0.7 nmol/L (+ AngII) after 24h. With corticosterone as substrate, Aldo production was 4.3 ± 1.1 nmol/L (no AngII) and 4.0 ± 0.9 nmol/L (+ AngII) respectively.

In H295R cells, Aldo baseline levels significantly increased upon Ang II stimulation with all substrates used (24h values; substrate progesterone: Aldo 0.4 ± 0.2 nmol/L (no Ang II), 2.0 ± 0.3 nmol/L (+ AngII); substrate DOC: Aldo 0.6 ± 0.2 nmol/L (no Ang II), 2.4 ± 0.2 nmol/L (+ Ang II); substrate corticosterone: Aldo 0.6 ± 0.2 nmol/L (no Ang II), 2.1 ± 0.7 nmol/L (+ AngII); substrate 18-OH-corticosterone: Aldo 0.8 ± 0.2 nmol/L (no Ang II), 1.1 ± 0.0 nmol/L (+ AngII)).

In JEG-3, BeWo, HTR-8/SVneo, HUVEC, HUAEC, HAEC, HRGEC, HLEC, HRMC, HEK293 cells, and in PBMCs of healthy subjects and PA patients no significant Aldo production could be detected in all conditions, except for the substrate 18-OH-corticosterone. In 18-OH-corticosterone supplemented JEG-3 and in all the other cell models (BeWo, HTR-8/SVneo, HUVEC, HUAEC, HAEC, HRGEC, HLEC, HRMC, HEK293, PBMCs of healthy subjects and PA patients) where 18-OH-corticosterone was used as substrate, there were low detectable baseline Aldo levels at the 0h time points and after 24h. As 24h levels were not significantly higher compared to the baseline levels, we assume no *de novo* production, but contribute these peak detections to contaminants in the 18-OH-corticosterone steroid standard stock.

### Progesterone metabolism and SRD5A1 mRNA expression

Progesterone levels decreased during the 24 h incubation period in JEG-3, BeWo, HTR-8/SVneo, HRMC, HEK293 cells, and in PBMCs of healthy subjects and PA patients; however, no relevant DOC, corticosterone, 18-OH-corticosterone, or Aldo levels could be detected. As progesterone metabolism was suspected to occur, 5α-reductase (*SRD5A1*) expression as well as the prominent formation of the progesterone metabolites: 6α/β-hydroxyprogesterone, 20α-hydroxyprogesterone, 11α-hydroxyprogesterone, 5α/β-dihydroprogesterone, allopregnanolone/isopregnanolone, and 6α-hydroxypregnanolone could be confirmed in JEG-3, BeWo, HTR-8/SVneo, HRMC, and HEK293 cells, and in PBMCs of healthy subjects and PA patients by real-time PCR and LC-MS analysis, respectively. Detailed results showing SRD5A1 ct values and absolute values of progesterone metabolites in nmol/L are shown in supplementary data (Supplementary Table 1, 2, 3, and 4).

Supplementary Fig. 2 shows an assumed progesterone metabolism pathway in JEG-3, BeWo, HTR-8/SVneo, HRMC, HEK293 cells, and in PBMCs of healthy subjects and hyperaldosteronism patients.

### mRNA expression of CYP21A2 in cells with active progesterone metabolism

CYP21A2 is the steroidogenic enzyme which converts progesterone to DOC in the adrenal glands. However, no DOC, and no metabolites downstream of DOC (corticosterone, 18-OH-corticosterone, and Aldo) were found in JEG-3, BeWo, HTR-8/SVneo, HRMC, HEK293 cells, and in PBMCs of healthy subjects and PA patients after supplementation with progesterone. These results therefore question the presence of CYP21A2 in these cell lines and the assessment of *CYP21A2* expression levels were additionally investigated. JEG-3, BeWo, HTR-8/SVneo, HRMC, HEK293 cells and the positive control H295R cells expressed significant levels of *CYP21A2*. No *CYP21A2* expression was however found in PBMCs of both cohorts and in HLEC cells. Cyclophilin A served as the endogenous control.

## Discussion

Neither significant *CYP11B2* mRNA expression nor *de novo* Aldo production from classical substrates was identified in various well-characterized, purchased, immortalized and primary human cell lines including mononuclear cells of healthy subjects and of patients suffering from PA with and without Ang II stimulation using a highly sensitive analytical method. The possibility that Aldo can be produced from mineralocorticoid intermediate steroid hormones downstream of progesterone was ruled out by supplying all assessed cells with DOC, corticosterone or 18-OH-corticosterone as steroid hormone substrates.

AngII, a known stimulator of the RAAS, was unable to boost *CYP11B2* expression and Aldo production in all the cells assessed. The positive controls (H295R cells and COS-7 cells overexpressing CYP11B2) expressed *CYP11B2* mRNA and produced Aldo. In H295R cells, Aldo production was stimulated up to five-fold by AngII, as expected. In BeWo, JEG-3, HTR-8/SVneo, HEK293, and HRMC cells, PBMCs and PBMCs of PA patients, progesterone levels decreased over time, but no classic downstream mineralocorticoid metabolites such as DOC, corticosterone, 18-OH-corticosterone or Aldo were detected. Progesterone metabolism such as it exists in many cells and organs (Lobo 1999, Klossner *et al.* 2021) was suspected to occur and could be confirmed. Even though JEG-3, BeWo, HTR-8/SVneo, HRMC and HEK293 cells express *CYP21A2*, they favor the conversion of progesterone to the downstream progesterone metabolites rather to DOC. Biological effects of the identified progesterone metabolites in off-target tissues are conceivable (Klossner *et al.* 2021).

In the positive controls, H295R cells, and COS-7/CYP11B2, 18-OH-corticosterone seems to be a suboptimal substrate for the CYP11B2; 18-OH-corticosterone’s conversion to Aldo was only marginal and not inducible by Ang II as compared to the substrates progesterone, DOC, and corticosterone. The very low expression of Ang II receptors in COS-7 could additionally explain this minor response. The finding of Reddish and Guengerich (Reddish & Guengerich 2019) that a higher enzyme concentration, more substrate, and more time are needed for the reaction 18-OH-corticosterone-Aldo to occur supports this assumption. The low Aldo levels found in several cell lines supplemented with high concentrations of 18-OH-corticosterone did not increase significantly with time or Ang II stimulation and therefore no Aldo was produced. A potential cross talk between 18-OH-corticosterone and Aldo could be ruled out as for the MS analysis, as Aldo was detected in negative ion mode at m/z 359.1864 at 5.47 min and 18-OH-corticosterone in positive mode at m/z 363.2166 at 6.64 min.

In any case, if such small Aldo concentrations hypothetically would be active, they would compete against a 1000× higher systemic concentration of cortisol – a steroid hormone with access to the MR in 11β-hydroxysteroid dehydrogenase 2-lacking off-target tissues (Ackermann *et al.* 2022).

### Findings in line with ours

In line with our results, no *CYP11B2* mRNA expression and Aldo biosynthesis was detected by the group of Gomez-Sanchez in three different human vascular endothelial cell lines, not even after stimulation with Ang II (Ahmad *et al.* 2004).

### Findings not in line with ours

Many research groups detected and published extra-adrenal *CYP11B2* expression and Aldo production in whole kidney tissue and/or renal cells (Wu *et al.* 1999, Nishikawa *et al.* 2005, Xue & Siragy 2005); in vessels and/or endothelial cells (Hatakeyama *et al.* 1994, Takeda *et al.* 1994, 1995*b*, 1996, 1997, Rudolph *et al.* 2000, Maron *et al.* 2012, 2014).

We assume, that the Aldo concentration found in several tissues comes from the adrenal glands and is not locally produced in these tissues or is erroneously detected. As Aldo sequestration was found in the brain (Gomez-Sanchez et al. 2010), it needs to be addressed, if adrenal Aldo can be stored, accumulated and released in off-target tissues.

### Strengths of our study

The strength of this study is the analysis of different steroid hormone metabolites with high resolution LC-MS. Utilizing H295R cells as a control cell line endogenously expressing functional CYP11B2 and the COS-7 cells transfected with the CYP11B2 plasmid supports our methodology. For our PCR analysis, amplification cycle number was 50, which is higher as in most studies performed and permits the detection of very low *CYP11B2* mRNA expression levels. Possible steroidogenic acute regulatory protein independent mechanisms were ruled out by adding steroid hormone substrates such as progesterone, DOC, corticosterone, and 18-OH-corticosterone.

### Limitations of our study

This study investigated *ex vivo* Aldo production in primary or immortalized, purchased human cell lines and self-isolated PBMCs of PA patients in 2D culture conditions and can therefore not be extrapolated 1:1 to *in vivo* conditions and tissues in which *de* *novo* Aldo production has been reported. As we did not analyze Aldo production in cells of all tissues mentioned to *de* *novo* synthesize Aldo in literature, we might have missed analyzing extra-adrenal tissues producing Aldo. But as Gomez-Sanchez *et al.* have correctly explained, there cannot be significant extra-adrenal Aldo production as in adrenalectomized animals no significant Aldo production was detectable, the amount of Aldo produced outside the adrenals is minimal and not clinically relevant (Ahmad *et al.* 2004). As some of the purchased primary cells and cell lines are from one single individual, gender, age-related, or intraindividual variability in these cells is possible. Other not yet characterized CYP11B2 substrates, cofactors, or stimulators are conceivable. Most of the assessed cell lines only marginally express AGTR1 and/or AGTR2 and therefore would not be expected to significantly respond to Ang II. *In vivo* studies investigating organ specific *de novo* Aldo production are complex and complicated by the systemic distribution of Aldo. Our protocol with incubation times of 24 h does not cover rapid mRNA changes or steroid hormone conversions. But if rapid mRNA changes were missed, steroid hormones would not be missed as they are stable for a long time once released.

### Summary

To summarize, no significant *CYP11B2* mRNA expression and no Aldo production could be detected in human vascular endothelial cell lines (HUVEC, HUAEC, HAEC, HRGEC), lymphatic endothelial cells (HLEC), in trophoblasts (BeWo, JEG-3, HTR-8/SV neo), in kidney cells (HEK293, HRMC), and in human peripheral blood mononuclear cells (PBMC) of healthy subjects and PA patients. If there is Aldo production in these cells, it is below detection limits of the LC-MS method and presumably not of clinical relevance.

We conclude that high circulating Aldo levels observed in PA patients are not due to Aldo production in PBMCs, nor are they due to autocrine/paracrine Aldo production in Aldo off-target tissues.

#### Supplementary materials

Supplementary Data

Supplementary Figure 1

Supplementary Figure 2

Supplementary Figure 3

Supplementary Figure 4

Supplementary Figure 5

Supplementary Figure 6

Supplementary Table 1

Supplementary Table 2

Supplementary Table 3

Supplementary Table 4

Supplementary Table 5

#### Declaration of interest

We declare that there is no conflict of interest that could be perceived as prejudicing the impartiality of the study reported.

#### Funding

This work was supported by the Swiss National Science Foundationhttp://dx.doi.org/10.13039/100000001 (personal Marie Heim-Vögtlin grant PMPDP3_151323 to CG-M and 32-135596 to MGM).
