# Supplementary material for: No extra-adrenal aldosterone production in various human cell lines
Source: J Mol Endocrinol. 2024 Feb 1;72(3):e230100. doi: 10.1530/JME-23-0100 (PMC10895282; doi:10.1530/JME-23-0100)

Supplementary Data to:

No extra-adrenal aldosterone production in various human cell lines

Isabelle Durrer[1], Daniel Ackermann[1], Rahel Klossner[1,2], Michael Grössl[1], Clarissa Vögel[1], Therina Du Toit[3,4], Bruno Vogt[1], Heidi Jamin[1,3], Markus G. Mohaupt [2,3], Carine Gennari-Moser[1,3].

[1]*Department of Nephrology and Hypertension; University of Bern, 3010 Berne, Switzerland*

[2]Department *of Internal Medicine, Sonnenhof, Lindenhofgruppe, Berne, Switzerland*

[3]*Department for BioMedical Research; University of Bern, 3010 Berne, Switzerland*

[4]*Division of Pediatric Endocrinology, Diabetology and Metabolism, Department of Pediatrics, Inselspital, Bern University Hospital, University of Bern, Switzerland*

Corresponding Author: Carine Gennari-Moser

Department of Nephrology and Hypertension

Freiburgstrasse 15

CH-3010 Berne, Switzerland

Email: carine.gennari@unibe.ch

Short title: Extra-adrenal aldosterone production

Keywords: CYP11B2, aldosterone, primary hyperaldosteronism, progesterone, cell lines

Methods

Real-time PCR

Cells were cultured for 24h in a steroid-free and phenol red-free medium alternative with or without Ang II (10^{-6} M). PBS was the solvent of Ang II and served as the baseline.

Extraction of total RNA was performed using the Trizol method. RNA was reverse transcribed by using Oligo dT and random hexamer in the same reaction (PrimeScript RT reagent Kit from TaKaRa). All RT experiments in all cell lines were performed the same way. 50 ng of cDNA was used for Real-time PCR. Assay on demand primers were used for human CYP11B2 (Hs01597732_m1), SRD5A1 (Hs 00971645_g1), CYP21A2 (Hs 00416901_g1), AGTR1 (Hs00258938_m1), AGTR2 (Hs02621316_s1), Cyclophilin A (*PPIA*, 4326316E) and 18S (4310893E). Cyclophilin A and 18S served as endogenous controls. They all were from Applied Biosystems (ThermoFisherScientific, Reinach, Switzerland). GoTaq Probe qPCR Master Mix A6102 was from Promega AG, Dübendorf, Switzerland.

H295R and COS-7 cells transfected with CYP11B2 were used as positive controls. Results are displayed as ct values. Amplification cycle number was 50 and assays were performed in triplicate.

7500 Fast Real-time PCR and Quant Studio 1 machine were used both for all cell lines assessed. They were from Applied Biosystems (Thermo-Fisher-Scientific, Reinach, Switzerland).

Liquid chromatography–mass spectrometry (LC-MS)

Cells were cultured for 24h in a steroid-free and phenol red-free medium alternative with the steroid hormone substrates progesterone, DOC, corticosterone or 18-OH-corticosterone at a concentration of 10^{-6}M and with or without Ang II (10^{-6}). EtOH was the solvent of the substrates and served as the baseline. Reasons for phenol red-

free medium were to exclude stimulatory conditions and interference of phenol red with the LC-MS equipment. After 24h cell supernatant was collected, centrifuged, aliquoted and stored at -20°C until LC-MS analysis.

For the LC-MS analysis, 500 µL cell aliquots were spiked with 38 µL internal standard mix and steroids subsequently extracted using solid-phase extraction on an OasisPrime HLB 96-well plate according to the protocol previously published (Andrieu et al., 2022). The LC-MS system consists of a Vanquish UHPLC (equipped with an ACQUITY UPLC HSS T3 Column, 100Å, 1.8 µm, 1 mm X 100 mm; Waters, Switzerland) coupled to a Q Exactive Orbitrap Plus (both from Thermo-Fisher-Scientific, Reinach, Switzerland). Separation was achieved using gradient elution over 17 minutes using water and methanol (mobile phase B) both supplemented with 0.1 % formic acid (all Sigma-Aldrich, Buchs, Switzerland) as mobile phases. The separation of steroid metabolites was achieved through the following elution gradient (at a constant flow of 0.15 mL/min): 0–0.5 min 1% B, 0.5–1 min linear gradient to 1–46% B, 1–4 min 46%, 4–12 min linear gradient 46–73% B, 12–12.5-min linear gradient 73–99% B, 12.5–14.5 min 99% B, 14.5–15-min linear gradient to 1% B, and 15–17 min 1% B. All LC-MS grade solvents required for analysis were from BioSolve (Switzerland).

Data analysis was performed using TraceFinder 4.1 (Thermo-Fisher-Scientific, Reinach, Switzerland).

Steroid hormone concentrations are displayed in nmol/L. The lower limit of accurate quantification (LLOQ) was 0.085 nmol/L for Aldo, 0.705 nmol/L for corticosterone, 0.476 nmol/L for progesterone and 0.092 nmol/L for DOC. 18-OH-Corticosterone was detected in the mass channel of corticosterone (m/z 347.2217), its elution time confirmed from timepoint 0h cell aliquots and it was quantified relative to the calibration curve of corticosterone.

For each batch of LC-MS analysis the same positive control H295R cells + AngII was used as internal control. The steroid hormone concentrations after 24h were compared to the initial baseline steroid hormone concentrations at timepoint 0h. Assays were performed in triplicate, except for HAEC and HRMC cells. HAEC and HRMC assays were performed only once due to material limits.

Primary hyperaldosteronism patients and healthy controls

Details of the purchased PBMCs of healthy volunteers are shown in Supplementary Figure 1.

Supplementary Figure 1

Cell Inventory

4W-270 - Human Peripheral Blood Mononuclear Cells (hPBMC)

24.11.2020

Material	Cell Type	Plant	Batch	Stock	Donor ID	Age	Sex	Race	Blood Type	Smoke	HIV/HCV/HBV	CMV	Viability [%]	Cell Count [in Million]
4W-270	hPBMC, 10M cells	US	3038013	34	11714	47	M	A	B+	No	Pass	Positive	95.0	17.0
4W-270	hPBMC, 10M cells	US	3038016	46	18424	21	M	C	A+	No	Pass	Negative	90.0	18.0
4W-270	hPBMC, 10M cells	US	3038019	16	18061	43	F	UNK	O+	No	Pass	Negative	90.0	14.0
4W-270	hPBMC, 10M cells	US	3038099	59	15211	23	M	C	B+	No	Pass	Positive	90.0	15.0
4W-270	hPBMC, 10M cells	US	3041652	20	20932	21	F	C	A+	Yes	Pass	Negative	95.0	13.0
4W-270	hPBMC, 10M cells	US	3041690	225	21600	44	M	C	O+	No	Pass	Negative	96.0	16.0

Cryopreserved ampule of Mononuclear Cell (MNC) rich cells from leukapheresis are depleted of RBCs and platelets.
Count and viability is determined using AO/PI. Cells are collected from healthy donors following IRB protocols. Manufactured by AllCells®

Results

Progesterone metabolism and SRD5A1 mRNA expression

Progesterone levels decreased during the 24h incubation period in JEG-3, BeWo, HTR-8/SVneo, HRMC, HEK293 cells, and in PBMCs of healthy subjects and PA patients, but no relevant DOC, corticosterone, 18-OH-corticosterone, and Aldo levels

could be detected. As progesterone metabolism was suspected to occur, 5α-reductase (*SRD5A1*) expression as well as the prominent formation of the progesterone metabolites 6α/β-hydroxyprogesterone, 20α-hydroxyprogesterone, 11α-hydroxyprogesterone, 5α/β-dihydroprogesterone, allopregnanolone and 6α-hydroxypregnanolone could be confirmed in JEG-3, BeWo, HTR-8/SVneo, HRMC, HEK293 cells, and in PBMCs of healthy subjects and PA patients by Real-time PCR and high resolution LC-MS-based methods, respectively. As isopregnanolone was only produced in HRMC, it is not displayed in all other tables. Detailed results showing *SRD5A1* ct values and absolute values of progesterone metabolites in nmol/L are shown in supplementary data **(Supplementary Table 1 and Supplementary Tables 2-4)**. LC-MS results are shown in absolute values nmol/L (mean ± SEM). The concentrations of the substrate progesterone at time point 0h and 24h are shown. The concentrations of all other progesterone metabolites are displayed as 24h values minus 0h values which reflects their true production. An unpaired parametric T-test was used to assess significance between 0h and 24h progesterone values. ND = not detected.

Supplementary Figure 2 shows an assumed progesterone metabolism pathway in placental and renal cells, and in PBMCs of healthy subjects and PA patients.

Supplementary Table 1: mRNA expression of *SRD5A1* and *CYP21A2* shown as ct values

Supplementary Figure 2

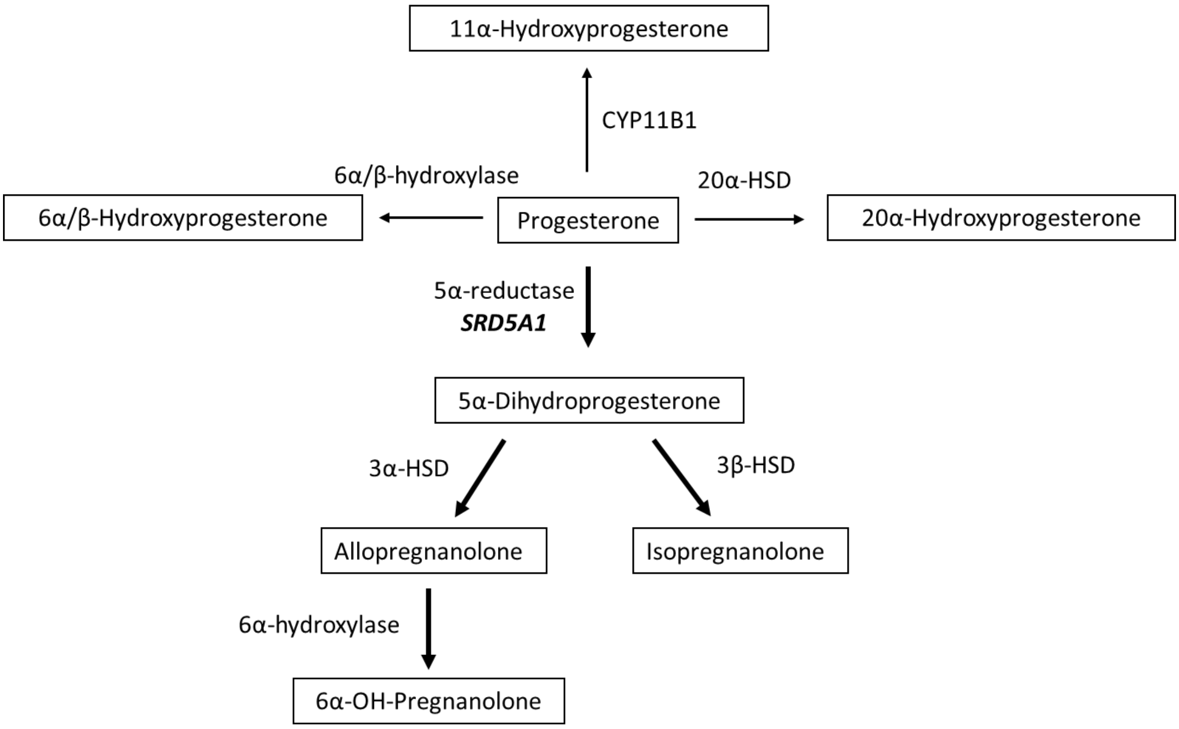

Supplementary Figure 2 legend: Assumed progesterone metabolism pathway in JEG-3, BeWo, HTR-8/SVneo, HRMC, HEK293 cells, and in PBMCs of healthy subjects and hyperaldosteronism patients.

mRNA expression of CYP21A2 in cells with active progesterone metabolism

CYP21A2 is the steroidogenic enzyme which converts progesterone to DOC in the adrenal glands. As no DOC, and no metabolites down-stream of DOC (corticosterone, 18-OH-corticosterone and Aldo) were found in JEG-3, BeWo, HTR-8/SVneo, HRMC, HEK293 cells, and in PBMCs of healthy subjects and PA patients after supplementation with progesterone, the presence of CYP21A2 needed to be assessed. JEG-3, BeWo, HTR-8/SVneo, HRMC, HEK293 cells and the positive control H295R cells expressed significant levels of *CYP21A2*. No *CYP21A2* expression was however found in PBMCs of both cohorts and in HLEC. Cyclophilin A served as the endogenous control.

Supplementary Table 1

mRNA expression of AGTR1 and AGTR2

JEG-3, HTR-8/SV neo, BeWo, HUVEC, HUAEC, HAEC, HLEC, HRGEC, HRMC, HEK293, H295R and COS-7/CYP11B2 cells were cultured as described above. RNA was isolated and real-time PCR was performed to detect mRNA levels of *AGTR1 and AGTR2*. Results are shown in Supplementary Table 5.

Supplementary Table 5

Progesterone metabolism measured by LC-MS

The prominent formation of the progesterone metabolites: 6α/β-hydroxyprogesterone, 20α-hydroxyprogesterone, 11α-hydroxyprogesterone, 5α/β-dihydroprogesterone, allopregnanolone/isopregnanolone and 6α-hydroxypregnanolone could be confirmed in JEG-3, BeWo, HTR-8/SVneo, HRMC, and HEK293 cells, and in PBMCs of healthy subjects and PA patients by LC-MS analysis.

Supplementary Table 2

Supplementary Table 3

Supplementary Table 4

Absolute values of the steroid hormone metabolites in PBMCs of healthy subjects and of PA patients supplemented with the substrates progesterone (Supplementary Figure 3), DOC (Supplementary Figure 4), corticosterone (Supplementary Figure 5), and 18-OH-corticosterone (Supplementary Figure 6) without (A, C) and with (B, D) Ang II shown as dot plots

Supplementary Figure 3

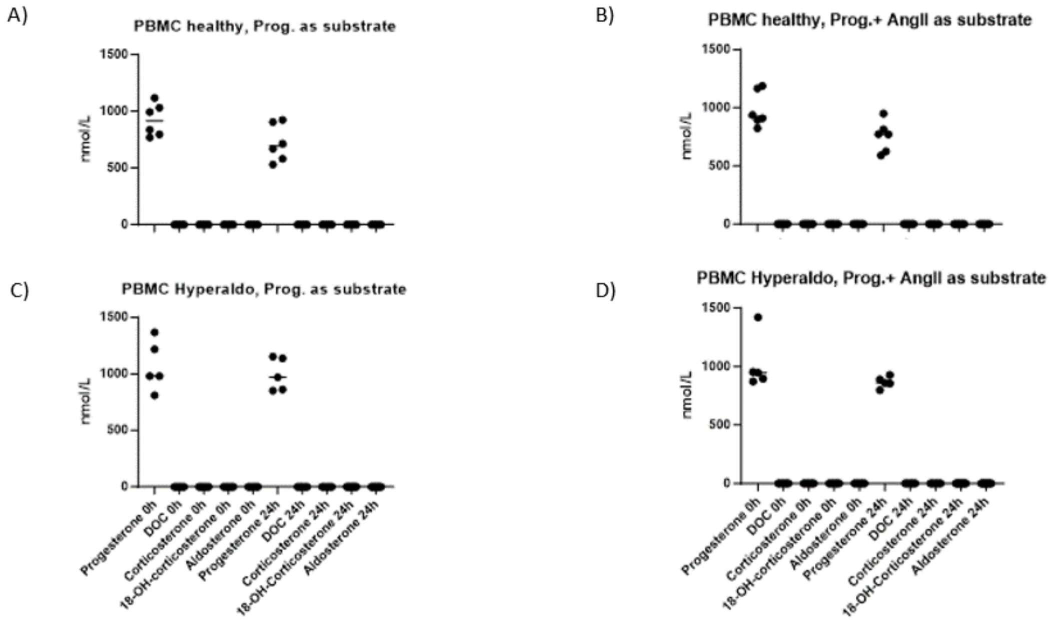

Supplementary Figure 4

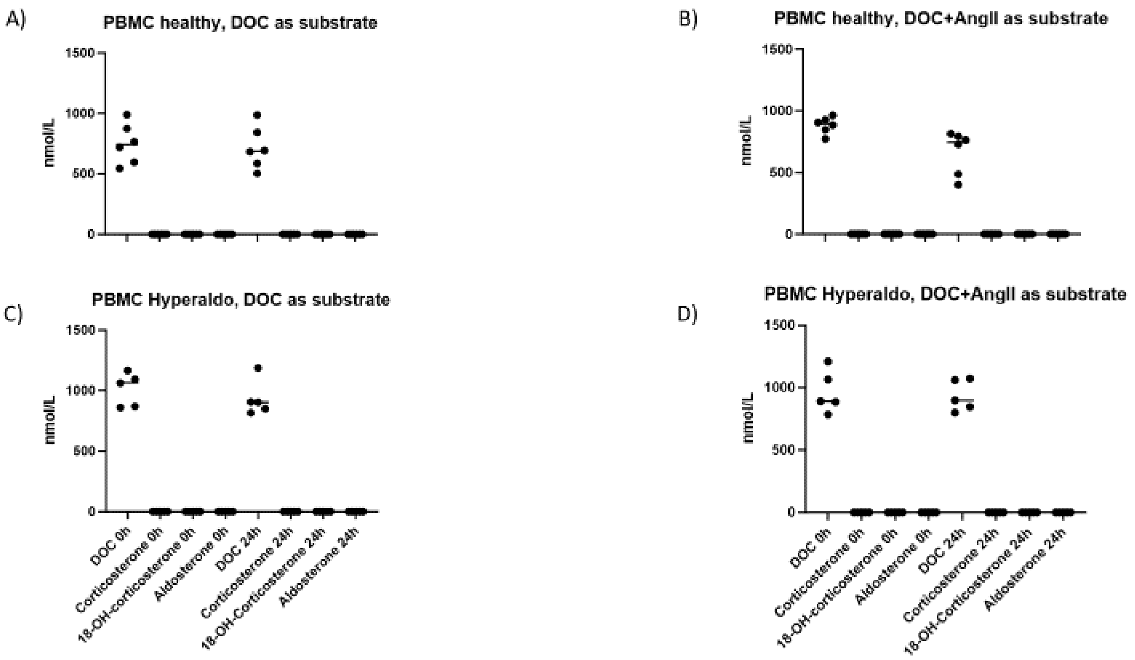

Supplementary Figure 5

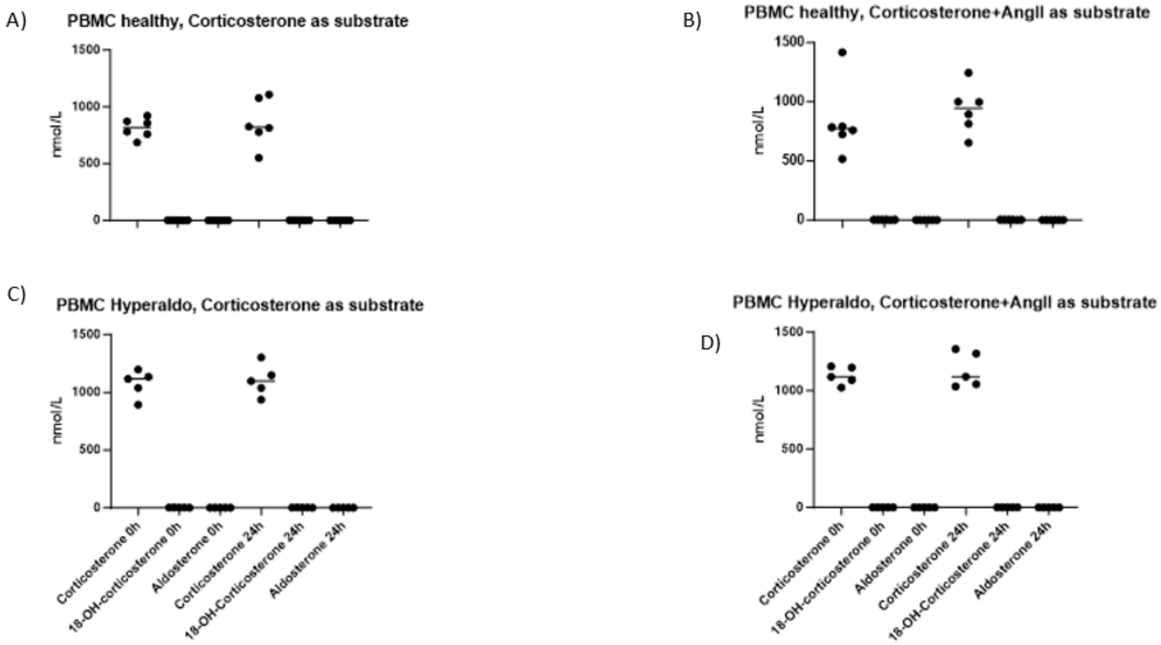

Supplementary Figure 6

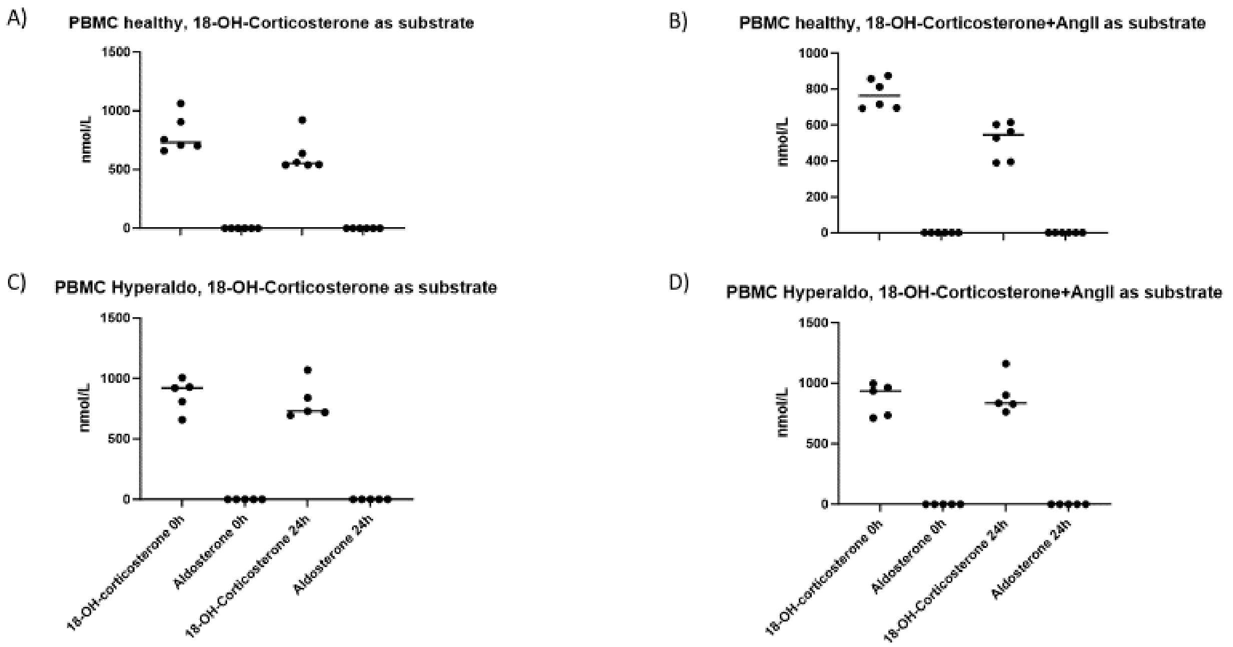

Supplement: Supplementary Data [file supplementary_data.pdf]
