# Supplementary material for: No extra-adrenal aldosterone production in various human cell lines
Source: J Mol Endocrinol. 2024 Feb 1;72(3):e230100. doi: 10.1530/JME-23-0100 (PMC10895282; doi:10.1530/JME-23-0100)
Supplement: Supplementary Figure 1 [file supplementary_figure_1.pdf]

Supplementary Figure 1

Cell Inventory

4W-270 - Human Peripheral Blood Mononuclear Cells (hPBMC)

24.11.2020

Material	Cell Type	Plant	Batch	Stock	Donor ID	Age	Sex	Race	Blood Type	Smoke	HIV/HCV/HBV	CMV	Viability [%]	Cell Count [in Million]
4W-270	hPBMC, 10M cells	US	3038013	34	11714	47	M	A	B+	No	Pass	Positive	95.0	17.0
4W-270	hPBMC, 10M cells	US	3038016	46	18424	21	M	C	A+	No	Pass	Negative	90.0	18.0
4W-270	hPBMC, 10M cells	US	3038019	16	18061	43	F	UNK	O+	No	Pass	Negative	90.0	14.0
4W-270	hPBMC, 10M cells	US	3038099	59	15211	23	M	C	B+	No	Pass	Positive	90.0	15.0
4W-270	hPBMC, 10M cells	US	3041652	20	20932	21	F	C	A+	Yes	Pass	Negative	95.0	13.0
4W-270	hPBMC, 10M cells	US	3041690	225	21600	44	M	C	O+	No	Pass	Negative	96.0	16.0

Cryopreserved ampule of Mononuclear Cell (MNC) rich cells from leukapheresis are depleted of RBCs and platelets.

Count and viability is determined using AO/PI. Cells are collected from healthy donors following IRB protocols. Manufactured by AllCells®
