# Supplementary material for: No extra-adrenal aldosterone production in various human cell lines
Source: J Mol Endocrinol. 2024 Feb 1;72(3):e230100. doi: 10.1530/JME-23-0100 (PMC10895282; doi:10.1530/JME-23-0100)

Supplementary Figure 5

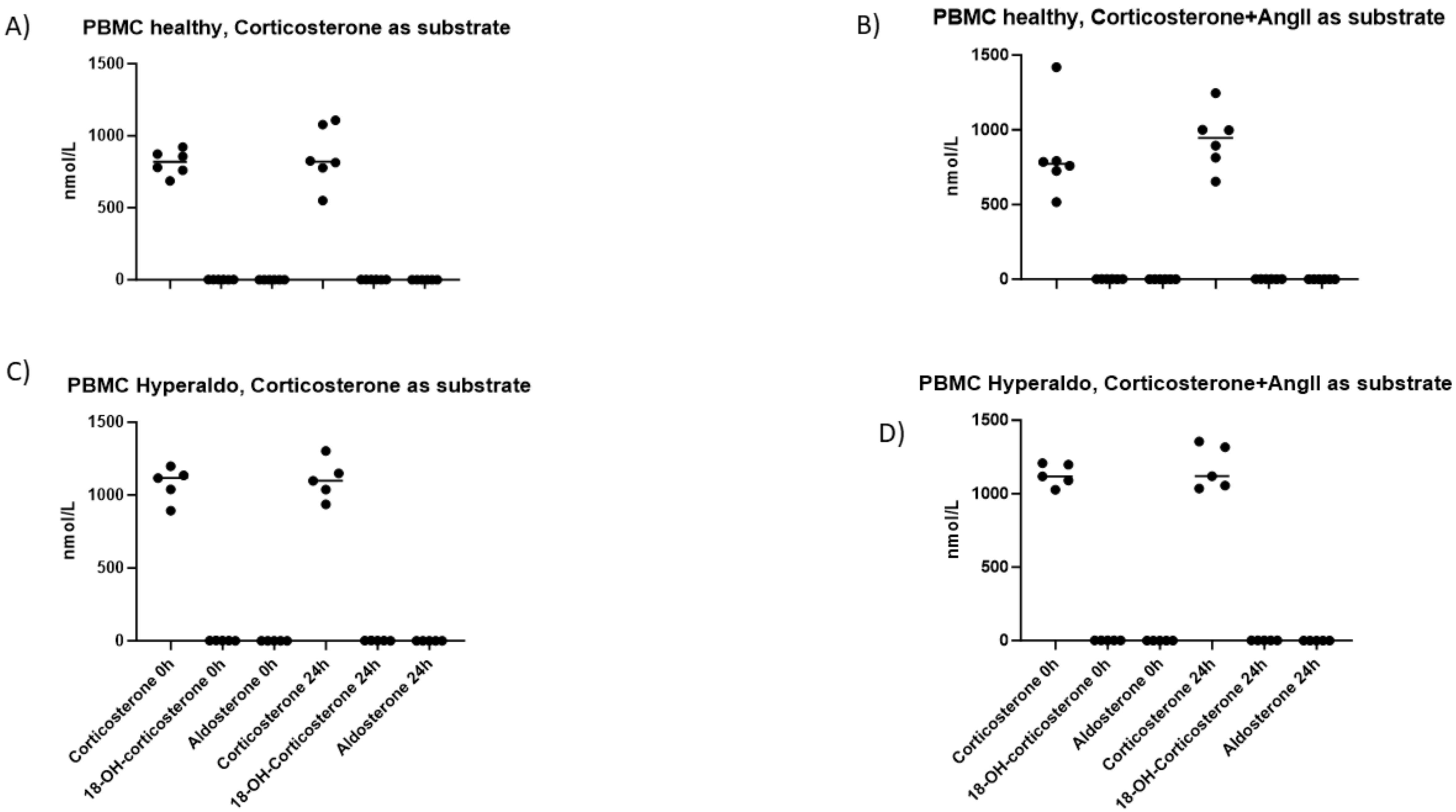

Supplement: Supplementary Figure 5 [file supplementary_figure_5.pdf]
