# Supplementary material for: No extra-adrenal aldosterone production in various human cell lines
Source: J Mol Endocrinol. 2024 Feb 1;72(3):e230100. doi: 10.1530/JME-23-0100 (PMC10895282; doi:10.1530/JME-23-0100)
Supplement: Supplementary Table 1 [file supplementary_table_1.pdf]

Supplementary Table 1

mRNA expression of SRD5A1 shown as ct values

	ct SRD5A1 no Ang II	ct SRD5A1 + Ang II
**JEG-3**	25.3	25.2
**BeWo**	26.3	26.7
**HTR-8/SV neo**	24.2	23.3
**HRMC**	23.8	23.5
**HEK293**	23.5	23.5
**PBMCs healthy subjects**	29.8	30.7
**PBMCs PA patients**	29.7	28.9
**HLEC**	28.0	28.0
**H295R**	23.9	23.9

mRNA expression of CYP21A2 shown as ct values

	ct CYP21A2 no Ang II	ct CYP21A2 + Ang II
**JEG-3**	31.6	31.6
**BeWo**	30.6	31.5
**HTR-8/SV neo**	34.0	32.0
**HRMC**	30.0	29.8
**HEK293**	30.7	30.5
**PBMCs healthy subjects**	> 35	> 35
**PBMCs PA patients**	> 35	> 35
**HLEC**	> 35	> 35
**H295R**	20.1	20.3
