# Supplementary material for: No extra-adrenal aldosterone production in various human cell lines
Source: J Mol Endocrinol. 2024 Feb 1;72(3):e230100. doi: 10.1530/JME-23-0100 (PMC10895282; doi:10.1530/JME-23-0100)
Supplement: Supplementary Table 5 [file supplementary_table_5.pdf]

Supplementary Table 5

mRNA expression of AGTR1 and AGTR2
cycle number: 50

Cell line	Condition	ct AGTR1	ct AGTR2
JEG-3	PBS	Undet	33.470
JEG-3	Ang II 10-6M	Undet	33.900
HTR-8/SV neo	PBS	35.985	Undet
HTR-8/SV neo	Ang II 10-6M	38.333	44.895
BeWo	PBS	Undet	35.264
BeWo	Ang II 10-6M	Undet	30.643
HUVEC	PBS	Undet	35.494
HUVEC	Ang II 10-6M	Undet	37.279
HUAEC	PBS	Undet	Undet
HUAEC	Ang II 10-6M	Undet	35.179
HAEC	PBS	38.501	32.895
HAEC	Ang II 10-6M	Undet	32.263
HLEC	PBS	Undet	31.687
HLEC	Ang II 10-6M	Undet	Undet
HRGEC	PBS	39.368	36.250
HRGEC	Ang II 10-6M	Undet	35.185
HRMC	PBS	34.473	33.892
HRMC	Ang II 10-6M	34.303	33.018
HEK293	PBS	34.444	33.026
HEK293	Ang II 10-6M	34.288	33.004
H295R	PBS	27.816	36.166
H295R	Ang II 10-6M	28.791	35.608
COS-7 + CYP11B2 plasmid	PBS	Undet	38.056
COS-7 + CYP11B2 plasmid	Ang II 10-6M	Undet	38.250

Undet: undetected
